# Fluid shear stress activates YAP to promote epithelial–mesenchymal transition in hepatocellular carcinoma

**DOI:** 10.1002/1878-0261.13061

**Published:** 2021-08-02

**Authors:** Hongchi Yu, Jia He, Guanyue Su, Yuelong Wang, Fei Fang, Wenxing Yang, Kaiyun Gu, Naiyang Fu, Yunbing Wang, Yang Shen, Xiaoheng Liu

**Affiliations:** ^1^ Institute of Biomedical Engineering West China School of Basic Medical Sciences & Forensic Medicine Sichuan University Chengdu China; ^2^ National Engineering Research Center for Biomaterials Chengdu China; ^3^ West China Hospital Sichuan University Chengdu China; ^4^ Department of Physiology West China School of Basic Medical Sciences & Forensic Medicine Sichuan University Chengdu China; ^5^ National Clinical Research Center for Child Health Zhejiang University Hangzhou China; ^6^ Cancer and Stem Cell Biology Program Duke‐NUS Medical School Singapore

**Keywords:** epithelial–mesenchymal transition, fluid shear stress, yes‐associated protein

## Abstract

Epithelial–mesenchymal transition (EMT) mediated by fluid shear stress (FSS) in the tumor microenvironment plays an important role in driving metastasis of the malignant tumor. As a mechanotransducer, Yes‐associated protein (YAP) is known to translocate into the nucleus to initiate transcription of genes involved in cell proliferation upon extracellular biophysical stimuli. Here, we showed that FSS facilitated cytoskeleton rearrangement in hepatocellular carcinoma cells, which led to the release of YAP from its binding partner, integrin β subunit, in the cytomembrane. Moreover, we found that upregulation of guanine nucleotide exchange factor (GEF)‐H1, a microtubule‐associated Rho GEF, is a critical step in the FSS‐induced translocation of YAP. Nuclear YAP activated the expression of the EMT‐regulating transcription factor *SNAI1*, but suppressed the expression of N6‐methyladenosine (m^6^A) modulators; together, this promoted the expression of EMT‐related genes. We also observed that FSS‐treated HepG2 cells showed markedly increased tumorigenesis and metastasis *in vivo*. Collectively, our findings unravel the underlying molecular processes by which FSS induces translocation of YAP from the cytomembrane to the nucleus, contributes to EMT and enhances metastasis in hepatocellular carcinoma.

## Graphical abstract

Here, we describe the underlying mechanism by which fluid shear stress (FSS) induces epithelial–mesenchymal transition (EMT) and enhances metastasis in hepatocellular carcinoma. Upon FSS, Yes‐associated protein (YAP) was released from its membrane binding partner integrin β subunits. Upregulation of GEF‐H1 promoted FSS‐induced nuclear translocation of YAP, which in turn regulated EMT‐related gene expression through *SNAI1* and m^6^A modulators.

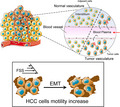

**Abbreviations**
E‐cad: E cadherin
EMT: epithelial–mesenchymal transition
FSS: fluid shear stress
FTO: fat mass and obesity‐associated protein
GEF: guanine nucleotide exchange factor
HCC: hepatocellular carcinoma
m^6^A: N6‐methyladenosine
METTL14: methyltransferase like 14
METTL3: methyltransferase like 3
N‐cad: N‐cadherin
TF: transcription factors
WTAP: Wilms tumor 1-associated protein
YAP: yes-associated protein 1

## 1 Introduction

The incidence and mortality of hepatocellular carcinoma (HCC) predominate in liver cancer, which is ranked at the sixth among the most commonly diagnosed cancer and the fourth among the leading causes of cancer death worldwide [1]. The high potential in invasiveness and metastasis is the primary cause of HCC‐related mortality. Increasing evidence showed that cancer invasion and metastasis are determined by the epithelial–mesenchymal transition (EMT), which is characterized by the loss of apicobasal polarity, and intercellular adhesion and acquisition of mesenchymal cell morphology and motility by cytoskeleton remodeling [2]. Biophysical factors in the tumor microenvironment, including stiffness and fluid pressure, have attracted much attention for their significant roles in determining cancer invasion and metastasis [3, 4]. The lymphoid and interstitial fluid moves over the solid tumor cells and generates laminar flow. The fluid shear stress (FSS), defined as the fluid frictional force between moving layers in laminar flow, acts as a direct mechanical stimulus to tumor cells. FSS has been identified as a contributing factor in promoting tumor cell motility in prostate cancer [5]. FSS exposure to the cells is approximately 0.1–2 dyn·cm^−2^ in a rich blood microvasculature of HCC microenvironment [6, 7]. However, the biomechanical mechanism of FSS‐induced EMT and its role in the high metastasis risk of HCC remain poorly understood.

Yes‐associated protein 1 (YAP) and its paralog, transcriptional co‐activator with PDZ‐binding motif (TAZ), bind primarily to the enhancer elements of their target genes using TEAD factors as DNA‐binding platforms [8]. Despite its widespread activation in tumors, it should be noted that no constitutively active YAP mutation has yet been reported in human cancers. The critical role of YAP in cancer development is mainly due to its increased shuttling between the cytoplasm and nucleus [9]. Recent reports indicated that a broad range of mechanical cues, including rigidity, tensional forces and stretching, could regulate YAP activation [10, 11, 12]. For instance, through the shear stress induced by the blood flow, YAP was activated to regulate cardiovascular development and disease [13, 14]. It has also been proposed that mechanical inputs from the tumor microenvironment could be the prime cue to induce YAP activation in tumor cells [15]. Integrins act as the most crucial mediator responding to mechanical stimuli, and their mechanobiological function is mainly controlled by quaternary structural changes [16]. Unidirectional shear stress was shown to activate integrins and promote integrin‐Gα13 interaction, which resulted in YAP phosphorylation and suppression through a signaling cascade in atherogenesis [17]. The EMT is mainly driven by multiple EMT‐associated transcription factors (EMT‐TF), including Twist1, Zeb1/2 and Snai1/2 [18]. However, the expression of EMT‐TFS alone is insufficient to initiate EMT, whereas they act as vital upstream EMT regulators. Although it is known that YAP can act as a mechanotransducer, how YAP is activated and its role in FSS‐induced EMT are poorly studied. Whether YAP is involved in the regulation of EMT‐TF transcription and other molecular events promoting the FSS‐induced EMT remains unclear.

Here, we showed that inhibition of YAP by shRNA led to the suppression of HCC metastasis and Rho‐GTPase activation. We provided evidence showing that the mechanotransduction process of YAP is tightly controlled by its physical interaction with integrin β subunit 1. The extracellular biomechanical signals induced rearrangement of cytoskeleton and separation of YAP from integrin β subunit 1. Moreover, guanine nucleotide exchange factor (GEF)‐H1 was identified as a critical mediator for the nuclear translocation of YAP in response to FSS. Activated YAP bound to the promoter region of SNAI1 (Snail) and directly initiated its transcription. In contrast, nuclear YAP directly acted on the promoter of N6‐Methyladenosine (m6A) modulators [(methyltransferase like 3 (METTL3), methyltransferase like 14 (METTL14), Wilms tumor 1‐associated protein (WTAP), and fat mass and obesity‐associated protein (FTO)] and suppressed their expression, which in turn reduced mRNA methylation of EMT‐related genes. Our findings suggest that FSS promotes EMT and metastasis of HCC through regulation of YAP activation, and we uncovered the important molecular events for this process.

## 2 Materials and methods

### 2.1 Human liver samples

Liver tumors and paired adjacent normal liver tissues were collected from West China Hospital, Sichuan University. The experiments were undertaken with the understanding and written consent of each subject and the study was approved by the medical review board of the West China Hospital of Sichuan University. The methods were carried out in accordance with the standards set by the Declaration of Helsinki.

### 2.2 Cell culture

All cell lines were purchased from the company (Shanghai Zhong Qiao Xin Zhou Biotechnology Co., Ltd., Shanghai, China) and were cultured under an atmosphere of 5% CO_2_ at 37 °C as described previously [19]. The human HCC cell lines (HCCLM3, HepG2 and Huh7) and LO2 were cultured in Dulbecco’s modified Eagle medium (DMEM; Gibco, Carlsbad, CA, USA) medium, PRIM‐1640 (Gibco), respectively. The medium was supplemented with 10% FBS (Gibco), penicillin (100 U·mL^−1^) and streptomycin (100 mg·mL^−1^).

### 2.3 Microfluidic devices and application of FSS

The fluid shear stress loading system and HepG2 cell loading conditions were described previously [19]. Cells were exposed to a laminar FSS (1.4 dyn·cm^−2^) for 2, 4 or 8 h, respectively. The loading shear stress was calculated according to the following formula [20, 21]:

$$\tau =\frac{6\mu Q}{WH^{2}}$$

where τ is the FSS loaded to the cells; μ is the viscosity of the circulating buffer; *Q* is the flow rate; *H* (0.3 mm) is the height of the chamber; and *W* (74.80 mm) is the width of the chamber. According to the above formula, when τ is 1.4 dyn·cm^−2^, the *Q* is 9.59 mL·min^−1^. The Reynolds number is 1444, indicating that the HCC cells were exposed to a laminar flow, which is calculated according to the following formula [22]:

$$\text{Re}=\frac{\rho VD}{\mu }$$

where ρ is the density of the circulating buffer; *V* is the velocity of the fluid; and *D* is the hydraulic diameter, which was calculated according to the following formula [23]:

$$D=\frac{2W^{\prime }H^{\prime }}{W^{\prime }+H^{\prime }}$$

where *W*′ is the width of cell‐seeded plate (48.63 mm) and *H*′ is the length of cell‐seeded plate (74.80 mm). Computational fluid dynamics (CFD) calculation was carried out according to the design of the flow chamber on the flat plate. FSS distribution in the contact surface with cells was analyzed. ADIND (ADINA R & D, Inc., Watertown, MA, USA) was used for three‐dimensional (3D) reconstruction, and the mesh of 3D geometry models was generated by a hexahedral. A total of 481 480 hexahedral elements of fluid are sufficient for this study, and the chamber reaches a sufficiently steady state after a loading flow of 5 s. The density and the viscosity of water and medium are: water at 37 °C: 993.3 kg·m^−3^ and 0.69 × 10^−3^ kg·m^−1^·s^−1^; DMEM medium with 10% FBS: 1009 kg·m^−3^ and 0.93 × 10^−3^ kg·m^−1^·s^−1^ [24]). The fluid velocity of the inlet and outlet is the boundary, the maximum volume of M_ASTER_FL_EX_®L/S® (Model 77200‐50, Masterflex, Vernon Hills, IL, USA) is 2300 mL·min^−1^, and the minimum volume is 0.06 mL·min^−1^.

### 2.4 Experimental animal model

Nude mice used in the present study were from (GPTech, Nanjing, China) and the animal study was approved by the Medical Ethics Committee of Sichuan University. Mice were kept at a constant temperature (21 ± 1 °C) under 12/12‐h light/dark cycle and had free access to water and standard chow. Male mice were used in all studies. For orthotopic transplantation, the mice were anesthetized by isoflurane inhalation and then injected with 50 μL of HepG2, HepG2 FSS, shYAP or shYAP FSS cell suspension at a concentration of 10^7^ mL^–1^. Mice were monitored until recovery in a chamber on a heating pad after surgery and fed a standard diet immediately after surgery until sacrifice. For abdominal tumor transplantation, mice were injected with 100 μL of HepG2, HepG2 FSS, shYAP or shYAP FSS cell suspension at a concentration of 10^7^ mL^–1^.

### 2.5 Cell migration and invasion assay

Wound healing assay were performed as described [19]; briefly, the cells were cultured on a sterile glass slide and allowed to form a confluent monolayer. A scratch wound was induced with a pipette tip. The glass slides were loaded into the FSS system. Images were acquired at different time points and analyzed using imagej software. Transwell invasion assays were performed as described [25]; cells (5 × 10^4^ cells per well) were seeded in the top chambers of the Transwell plates in FBS‐free media with membrane inserts coated with Matrigel. After incubation for 24 h, the cells that had invaded the lower surface of the membrane were fixed, stained with crystal violet, and observed using an inverted microscope.

### 2.6 Western blotting

Western blotting was conducted as described previously [19]. After exposure to FSS, the cells were washed twice with PBS on ice and homogenized in cold RIPA lysis buffer containing 1% PMSF and Complete™ protease inhibitor cocktail (Roche Molecular Biochemicals, Indianapolis, IN, USA). The protein concentration was determined using Bradford Assay (Bio‐Rad, Hercules, CA, USA). A 20‐mg aliquot of protein was loaded in SDS/PAGE and transferred to the poly(vinylidene difluoride) (PVDF) membrane (cat. number‐IPVH00010, Millipore, Darmstadt, Germany). Primary antibodies were diluted in TBST containing 5% BSA or 3% nonfat‐milk to detect target protein. All experiments were carried out more than three times, and the target proteins were quantified by the Image lab (Bio‐Rad) and normalized to internal control. Nuclear and cytoplasmic extracts were prepared using the NE‐PER Nuclear and Cytoplasmic Extraction kit (Thermo Fisher Scientific, Inc., Waltham, MA, USA), according to the manufacturer’s instructions. Detailed information on all antibodies used in this study is provided in Table S1.

### 2.7 Immunofluorescence

Cells were fixed in 4% paraformaldehyde for 15 min at room temperature (RT) as previously described [20]. After washing three times in PBS, the cells were permeabilized by 0.1% Triton X‐100 and blocked by 5% goat serum in PBS for 30 min at RT. The cells were incubated with primary antibodies overnight at 4 °C and washed at RT for 90 min. The washed samples were then incubated with secondary antibodies diluted in PBS containing 5% goat serum. The nuclei were stained with DAPI for 10 min at RT. Five randomly selected views were selected to observe the expression of target proteins.

### 2.8 TEM

Transmission electron microscope (TEM) observation was performed as previously described [19], samples were treated by shear stress and gently collected using a plastic cell scraper (Corning, NY, USA) to maintain the origin cell–cell junction. The samples were centrifuged at 1800 ***g*** for 10 min, fixed with 0.5% glutaraldehyde, and stored at 4 °C for 10 min. Then, the samples were centrifuged again at 20 000 ***g*** for 10 min, the supernatant was discarded, and 3% glutaraldehyde was added slowly. All samples were fixed with 1% OsO_4_, dehydrated by different concentrations of acetone, embedded in epoxy resin (Epon812, TAAB Laboratories Equipment Ltd., Berks, UK), cut into slices, and double‐dyed using uranyl acetate and lead citrate. The microstructure of cell–cell junctions was observed by TEM (H‐600IV, Hitachi, Ltd., Tokyo, Japan).

### 2.9 Immunoprecipitation and immunoblotting

Immunoprecipitation and immunoblotting were performed as previously described [26]. Cells were lysed in RIPA buffer for 30 min on ice and then centrifuged (12 000 ***g***, 10 min, 4 °C). Lysates containing 500 mg of protein were incubated with a pull‐down antibody overnight at 4 °C. A/G beads (cat. number–P2012, Beyotime Institute of Biotechnology, Shanghai, China) were added, and the mixture was incubated with rotation for 2 h at 4 °C. Beads were washed three times in RIPA buffer, centrifuged at 5000 ***g*** for 1 min at 4 °C, and then boiled with loading buffer and a supernatant taken. Using 10% SDS/PAGE (100 V, 90 min), 20–35 mg of the protein extract was separated and transferred onto a PVDF membrane (cat. number‐IPVH00010, Millipore) at 30 V for 1.5 h. The PVDF membrane was blocked in 5% BSA (cat. number‐0332, Amresco, Atlanta, GA, USA) dissolved in TBS‐T (10 mm Tris‐HCl, pH 7.5, 150 mm NaCl and 0.1% Tween 20) for 1 h at RT. Blots were then incubated overnight at 4 °C in TBS‐T containing 5% BSA and a primary antibody at a 1 : 500 dilution. After three washes in TBS‐T at RT, appropriate secondary‐horseradish peroxidase (HRP) antibodies (cat. number‐ZB2301, ZSGB‐BIO, Beijing, China) were applied at a dilution of 1 : 5000 for 1 h at RT in TBS T containing 5% milk. Blots were washed in TBS‐T with HRP activity being detected by ECL (cat. number‐P1008, Beyotime Institute of Biotechnology).

### 2.10 Cytoskeletal protein F‐actin staining

Cells were washed with PBS and fixed with 4% paraformaldehyde for 15 min and incubated with phallotoxin (Thermo Fisher Scientific, Inc.) for 1 h. DAPI (4′, 6′‐diamidino‐2‐phenylindole) with 1 : 800 dilution was added to the samples and incubated for 30 min at 37 °C. All samples were examined by laser scanning confocal microscopy as described previously [26] (TCS SP5, Leica, Wetzlar, Germany).

### 2.11 Pull‐down assay/Rac1 activity assay

GST‐human Pak1‐PBD (Thermo Fisher Scientific, Inc.) was used for the Rac1 activity assay according to the manufacturer’s instruction [26]. Cells were washed three times with PBS and then lysed in lysis buffer. After centrifugation at 16 000 ***g*** for 15 min at 4 °C, the supernatants (containing 1 mg of total proteins) were collected and incubated with GST‐human Pak1‐PBD on agarose beads for 1 h with gentle rocking at 4 °C. The beads were collected by centrifugation at 6000 ***g*** for 30 s and washed three times with lysis buffer. Then, 2 × SDS sample buffer was used to elute the immobilized proteins on beads by boiling for 5 min. The samples were subjected to western blot analysis and the protein expression levels were detected with an anti‐Rac1 antibody.

### 2.12 Immunohistochemical staining

Tumor tissues were fixed in formalin and embedded in paraffin blocks using standard methods described previously [19]. Immunohistochemistry was used to detect target proteins. HRP‐conjugated secondary antibodies were used. Blocking and chromogenic detection were performed using the Dako Envision System with DAB substrate (Agilent, Santa Clara, CA, USA) according to the manufacturer’s protocol. Primary antibodies used in this study are listed in Table S1.

### 2.13 Gene expression profiling

Total RNA from each sample was extracted using TRIzol reagent (Invitrogen) as per the manufacturer’s instructions. Next‐generation sequencing was performed by Aksomics, Inc. (Shanghai, China) [27]. Briefly, after agarose gel electrophoresis for quality control and NanoDrop check for quantification, total RNA was used to prepare the sequencing library. The quality control of the library was conducted by Agilent 2100 (Agilent) and quantified by qPCR. Sequencing was performed by Illumina Hiseq 4000 (Illumina, San Diego, CA, USA). Sequencing quality was examined using fastqc software (Babraham Institute, Cambridge, UK). The transcript abundances for each sample were estimated with StringTie, and the FPKM value (≥ 0.5) for gene and transcript levels was calculated with r package Ballgown (version 2.10.0). The differentially expressed genes and transcripts were filtered using r package Ballgown. The correlation analysis was based on gene expression levels (principal component analysis). Hierarchical Clustering, Gene Ontology (GO) and Pathway Analysis were performed based on the differently changed genes.

### 2.14 Label‐free quantitative proteomics

Membrane and nuclear fractions of the HepG2 cells were extracted after FSS loading using Mem‐PER™ Plus Membrane Protein Extraction Kit and NE‐PER™ Nuclear and Cytoplasmic Extraction Reagents (Thermo Fisher Scientific, Inc.) following the instructions. After Co‐IP with YAP antibody, protein samples were washed out and prepared for LC–MS/MS analysis. The raw data from LC–MS/MS analysis were processed with maxquant software (version 1.5.6.0, Max Planck Institute of Biochemistry, Martinsried, Germany) [28]. The GO functional analysis was performed for differential expression of proteins.

### 2.15 ChIP‐qPCR

ChIP‐qPCR was performed as described previously [8]. Briefly, 100 μg of sheared chromatin and 5 μg of antibody were used. Quantitative real‐time PCR was carried out with a Rotor‐GeneQ (Qiagen, Manchester, UK) thermal cycler. Each sample was analyzed in triplicate.

### 2.16 ChIP‐qPCR data analysis

Normalizing the Ct value of each ChIP DNA fraction to the Input DNA fraction Ct value for the same qPCR Assay (ΔCt) was used to represent chromatin sample preparation differences. The % Input of each ChIP fraction was calculated using the following formula:

%Input = 2^(CtInput − CtChIP)^ × Fd × 100%

where Fd is the input dilution factor (in this study 1/5); fold enrichment = [%(ChIP/Input)]/[%(Negative control/Input)]; the normalized ChIP fraction Ct value was adjusted for the normalized background (mock IP) fraction Ct value; ΔΔCt [ChIP/mock IP] = ΔCt [normalized ChIP] − ΔCt [normalized mock IP]. PCR primers are listed in Table S2

### 2.17 Quantitative RT‐PCR

The method performing qPCR was described previously [19] and gene expression was normalized by GAPDH or β‐actin. All primers used in the present research are shown in Table S3. The relative fold change in RNA expression was calculated using the 2^−ΔΔCt^ method.

### 2.18 Methylation microarray

Total RNA from each sample was quantified using the NanoDrop ND‐1000. The sample preparation and microarray hybridization were performed based on Arraystar standard protocols [29]. Briefly, the total RNA was immunoprecipitated with anti‐N6‐methyladenosine (m^6^A) antibody. The modified RNAs were eluted from the immunoprecipitated magnetic beads as ‘IP’. The unmodified RNA was recovered from the supernatant as ‘Sup’. The ‘IP’ and ‘Sup’ RNA were labeled with Cy5 and Cy3 respectively as cRNA in independent reactions using the Arraystar Super RNA Labeling Kit. The cRNA were combined and hybridized onto Arraystar Human mRNA & lncRNA Epitranscriptomic Microarray (8x60K, Arraystar, Rockville, MD, USA). After washing the slides, the arrays were scanned in two‐color channels using an Agilent Scanner G2505C.

### 2.19 Microarray data analysis

agilent feature extraction software (version 11.0.1.1) was used to analyze acquired array images. Raw intensities of IP (immunoprecipitated, Cy5‐labeled) and Sup (supernatant, Cy3‐labeled) were normalized with an average of log_2_‐scaled spike‐in RNA intensities. After spike‐in normalization, the probe signals having Present (P) or Marginal (M) QC flags in at least one of two samples were retained as ‘All Targets Value’ in the excel sheet for further ‘m^6^A quantity’ analyses. The m^6^A quantity was calculated for the m^6^A methylation amount based on the IP (Cy5‐labeled) normalized intensities. Differentially, m^6^A‐methylated RNA between two comparison samples was identified by filtering with the fold change. Hierarchical clustering was performed using r software. GO analysis was performed using the top GO package in r environment for statistical computing and graphics, and Pathway Analysis was calculated using Fisher’s exact test [29].

### 2.20 Actin segmentation by ultracentrifugation

Different fractions of actin proteins were separated by ultracentrifuge according to the protocol [30]. In brief, diluted cell lysates with equal volumes were used for fractionation; another volume was kept separately as ‘total protein inputs’. The F‐actin and G‐actin pools of the diluted cell lysates were separated by ultracentrifugation at 100 000 ***g*** for 1 h at 37 °C. After centrifugation, F‐actin sediments can be resuspended in cold distilled water with 1 mm cytochalasin D (Sigma‐Aldrich, Darmstadt, Germany) and kept on ice for 45 min, whereas G‐actin remains in the supernatant. Laemmli buffer was added to both fractions and ‘total protein inputs’ before being boiled and analyzed by western blotting.

### 2.21 Plasmid construction, lentiviral production and transfection

To generate YAP knockdown cells, oligonucleotides were cloned into psi‐LVRU6GP with the *Bam*HI/*Eco*RI sites. The sequences of the oligonucleotides are as follows: YAP‐sense, 5′‐TAATACGACTCACTATAGGG‐3′; YAP‐antisense, 5′‐CTGGAATAGCTCAGAGGC‐3′. Four YAP1 shRNA targeting different sequences were designed, here referred to as shYAP21, shYAP22, shYAP23, shYAP24, respectively. Empty vector was used as a control, referred to as shTR001. The target sequences are listed as Table S4. Plasmids were transformed, propagated and purified from Stbl2 competent cells. To establish a HepG2 cell line with YAP stably silenced, HEK293‐T cells were co‐transfected with viral plasmid and lentiviral packaging plasmids psPAX2 and pMD2.G. The medium after 48 and 72 h transfection was collected and filtered through a 0.4‐μm filter. Subsequently, the medium containing lentiviral particles was centrifuged at 5000 ***g*** for 30 min, and the PEG precipitation was resuspended with serum‐free DMEM medium (Gibco), which was then used to infect HepG2 cells. Cells were screened by high glucose DMEM culture medium containing 0.5 μg·mL^−1^ puromycin. To generate luciferase and shYAP co‐transfected cells, we transfected the indicated plasmids (Genechem, Shanghai, China) containing the luciferase gene in the cells with stably silenced YAP gene.

### 2.22 Statistical analysis

Statistical analyses were performed using graphpad prism v5.02 for Windows (GraphPad Software, San Diego, CA, USA). Statistical significance was determined using one‐way analysis of variance (ANOVA) followed by Tukey’s‐test or two‐tailed unpaired *t*‐test. At least three independent experiments were performed for all biochemical experiments, and the representative images were shown. Results represent mean ± SEM.

## 3 Results

### 3.1 FSS promotes EMT of HCC cells

**Fig. 1:**
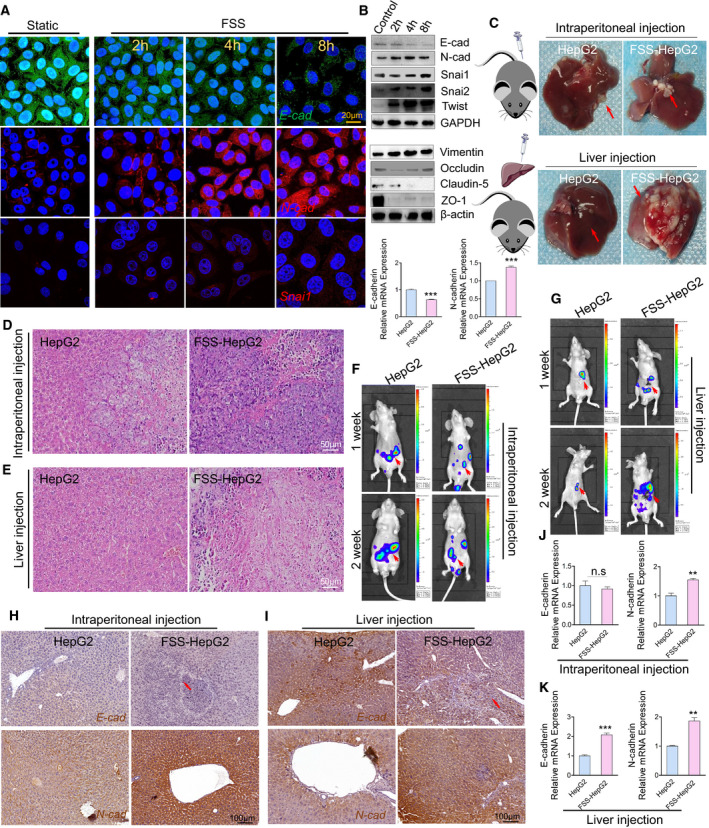
FSS induces EMT in HepG2 cells. (A) Immunostaining illustrating the location and expression of E‐cad (green), N‐cad (red) and Snai1 (red) in HepG2 subjected to FSS. Nuclei were counterstained with DAPI (blue). Scale bar: 20 μm. (B) Western blotting and qPCR analysis of proteins/genes involved in EMT following FSS treatment. GAPDH and β‐actin were used as internal controls (*n* = 3). (C) Representative images of the liver specimens from nude mice at 2 weeks after intraperitoneal or orthotopical injection of statically cultured HepG2 cells or FSS‐HepG2 cells. The arrows indicated the tumorigenesis. (D,E) HE staining of the liver tumor slices from the mice 2 weeks after intraperitoneal and orthotopic injection. Scale bar: 50 μm. (F,G) Live imaging of nude mice 2 weeks after injection of either statically cultured HepG2 cells or FSS‐HepG2 cells transfected with luciferase. The arrows indicated the tumor metastasis. (H) Representative images showing immunohistochemical staining of E‐cad and N‐cad from mice injected intraperitoneally with either static cultured HepG2 or FSS‐HepG2 cells. The arrows indicate the expression of E‐cad. (I) Representative images showing immunohistochemical staining of E‐cad and N‐cad from mice implanted orthotopically with either static cultured HepG2 or FSS‐HepG2 cells. (J,K) qPCR analysis of the EMT marker genes from the liver tissues of the mice with intraperitoneal and orthotopical injection of either statically cultured HepG2 or FSS‐HepG2 cells (*n* = 3). Data are presented as mean ± SEM. Statistics was performed by two‐tailed unpaired *t*‐test: **P < 0.01, ***P < 0.001, n.s. no significance.

Epithelial–mesenchymal transition is a multistep biological process (BP) in the initial stage of tumor metastasis [31]. The EMT status is accompanied with increased motility of tumor cells [2, 32]. We found that, compared with the non‐transformed human fetal hepatocyte cell line LO2, the HCC cell line HepG2 showed lower expression of epithelial marker E‐cadherin (E‐cad) and higher expression of mesenchymal marker N‐cadherin (N‐cad; Fig. S1A,B and Fig. 1C, upper panel). Rho‐family GTPase, including RHOA, RAC1 and CDC42, are involved in the dynamics of stress fibers, lamellipodia and filopodia and play a crucial role in cell motility [33]. In comparison with LO2 cells, HepG2 cells showed faster migration (Fig. S1D) and a well‐organized cytoskeleton (Fig. S1E). In line with this, HepG2 cells exhibited higher expression levels in RAC1, RHOA and CDC42 (Fig. S1F–I). These results suggest that HCC cells have EMT status and enhanced motility.

To evaluate the effects of FSS of the fluid microenvironment on HCC, we set up a parallel plate chamber to produce FSS with defined parameters on HepG2 cells as described previously [19]. In the area that is in direct contact with cells, the Reynolds number (1444) was lower than 2000, confirming a laminar flow in the parallel plate chamber. The distribution analysis of FSS showed that the average FSS on cells is approximately 1.4 dyn·cm^−2^ (Fig. S2A–E). Confocal imaging analysis indicated that the E‐cad signal gradually declined in the cells with increased duration of exposure to FSS. In contrast, FSS induced a rapid increase of the N‐cad expression (Fig. 1A). EMT‐TF, including Snai1/2 and Twist1, are known to play an important role in driving EMT [18]. Confocal imaging analysis also showed that the Snai1 expression was elevated in the HepG2 cells subjecting to FSS (Fig. 1A). This was further confirmed by western blotting and qPCR analysis (Fig. 1B, upper panel and lower panel). Moreover, FSS induced the expression of the mesenchymal marker vimentin [34, 35] but suppressed the expression of the tight junction proteins Occludin, Claudin‐5 and ZO‐1, suggesting that FSS promoted the invasive ability and diminished intercellular connections (Fig. 1B, middle panel). We tested the effects of EMT induction by FSS on another two human HCC cell lines (HCCLM3 and Huh7) and the non‐transformed liver cell line LO2 (Figs S1J and S3A,B). As observed in HepG2 cells, FSS induced HCCLM3 and Huh7 cells to undergo EMT with decreased E‐cadherin and increased N‐cadherin. Interestingly, the LO2 cells did not show significant change in the expression of EMT markers tested, suggesting that FSS is only able to accelerate the EMT process in HCC cells, not in non‐transformed cells.

To investigate whether the FSS‐induced EMT status in the cells could be maintained *in vivo*, control HepG2 cells and HepG2 cells treated with FSS (FSS‐HepG2 cells) were delivered to nude mice immediately after FSS exposure by either intraperitoneal or orthotopic injection. Two weeks after injection, liver tissues from the mice injected with the FSS‐HepG2 cells showed markedly increasing tumorigenesis and invasion (Fig. 1C–G). In line with this, IHC staining revealed that the expression of E‐cad and N‐cad markedly decreased and increased, respectively, in the injected FSS‐HepG2 cells (Fig. 1H,I). EMT‐associated TF Snai1 and tumor invasive marker MMP‐9 [36] showed elevated expression in the FSS‐HepG2 cells (Fig. S3C,D). The mRNA levels of *E‐cad* and *N‐cad* were also significantly different in the livers from mice injected with control HepG2 and FSS‐HepG2 cells (Fig. 1J,K).

**Fig. 2:**
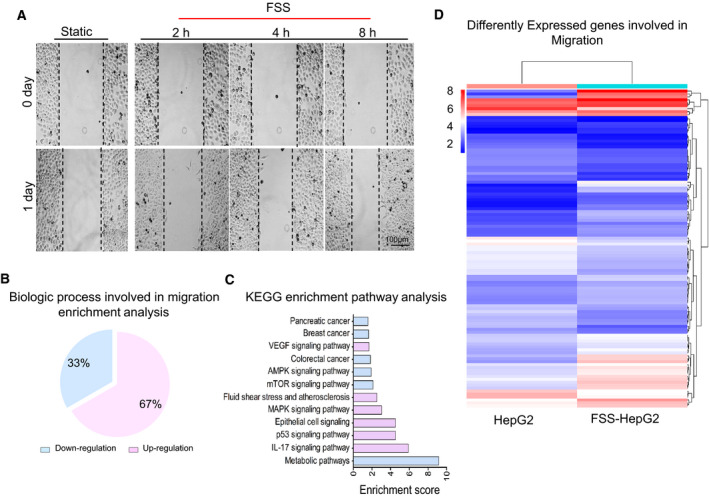
FSS accelerates HepG2 cell migration. (A) FSS enhances the migration ability of HepG2 in wound healing assay. Scale bar: 100 μm. (B) GO enrichment analysis for the mRNA profile involved in migration. (C) KEGG pathway enrichment analysis for the FSS‐HepG2 cells compared with statically cultured HepG2 cells. Up‐ and downregulation are shown in red and blue, respectively. (D) Heatmap showing differently expressed genes involved in migration and identified by RNA‐seq in the comparison of static HepG2 cells with FSS‐HepG2 cells.

A wound‐healing assay was conducted to examine whether FSS could promote the migration of HepG2 cells. We found that the motility of HepG2 cells was enhanced by FSS and positively correlated with exposing duration (Fig. 2A). To identify the potential genes and pathways involved in FSS‐induced EMT and cell migration, we conducted global gene expression profiling on control and FSS‐treated HepG2 cells. Interestingly, BP enrichment analysis revealed that FSS‐induced genes were associated with cell motility function. The ratio of upregulated BP terms involved in migration accounted for 67% (Fig. 2B). KEGG enrichment analysis revealed 12 enriched pathways, including FSS and epithelial cell signaling (Fig. 2C). Notably, 108 differently‐expressed genes (*P* < 0.05 and absolute fold change ≥ 1.5) involved in cell migration were identified (Fig. 2D, Table S5). These results together provided strong evidence suggesting that FSS, as a biophysical stimulus, facilitated the EMT and cell mobility in HCC cells.

### 3.2 FSS induces the nuclear accumulation of YAP

**Fig. 3:**
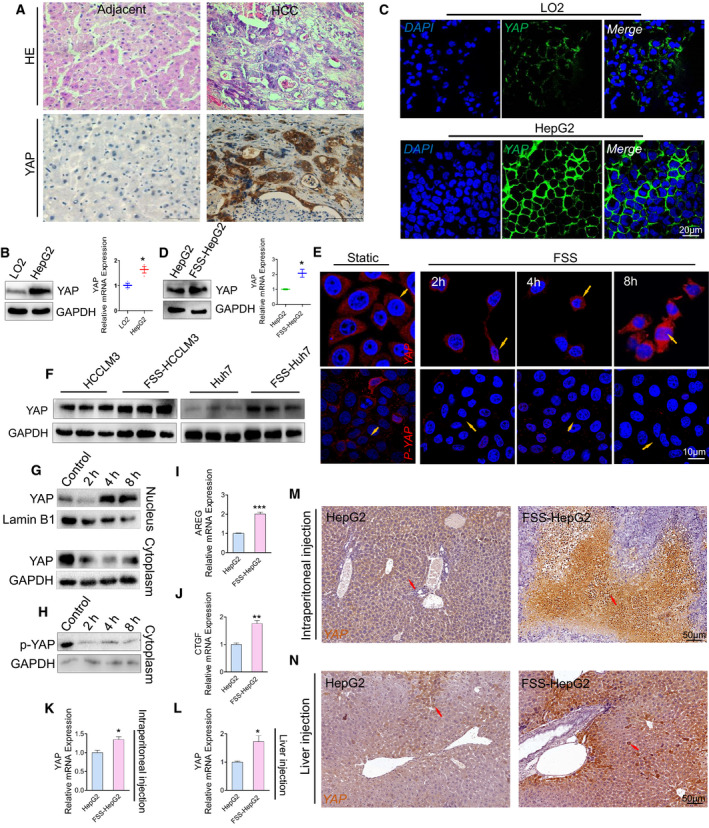
Nuclear accumulation of YAP induced by FSS. (A) Representative images showing immunohistochemical staining of YAP in human HCC tumors (*n* = 3). Scale bar: 50 μm. (B) Western blotting analysis and qPCR quantification of *YAP*, *GAPDH* used as internal control (*n* = 3). (C) Immunostaining illustrating the location and expression of YAP in HepG2 cells and LO2 cells. Scale bar: 20 μm. (D) Western blotting analysis and qPCR of YAP in FSS‐HepG2 cells. GAPDH is used as internal control. (E) Immunostaining showing that FSS promoted the nuclear translocation of YAP and the nuclear export of p‐YAP in HepG2 cells. The arrows indicated the nuclear location of YAP. Scale bar, 10 μm. (F) Western blotting analysis of YAP in indicated control HCC and FSS‐HCC cells. GAPDH was used as internal control. (G) Western blot analysis of YAP in nuclear and cytoplasmic fractions in HepG2 and FSS‐HepG2 cells. Lamin B1 and GAPDH were used as internal control for nuclear and cytoplasm, respectively. (H) Western blotting analysis of p‐YAP in the cytoplasmic fraction in HepG2 and FSS‐HepG2 cells. GAPDH was used as internal control. (I, J) qPCR analysis of the YAP target genes *AREG* and *CTGF* in HepG2 and FSS‐HepG2 cells (*n* = 3). (K, L) qPCR analysis of *YAP* in the liver specimens obtained from mice with intraperitoneal or orthotopic injection (*n* = 3). (M, N) Representative images showing immunohistochemical staining of YAP in the liver tissues of the mice intraperitoneally or orthotopically injected with static cultured HepG2 or FSS‐HepG2 cells. The arrows indicated the nuclear location of YAP. Scale bar, 50 μm. Data are presented as mean ± SEM. Statistics was performed by a two‐tailed unpaired *t*‐test. *P < 0.05, **P < 0.01, ***P < 0.001.

We next addressed the underlying biomechanical mechanisms by which FSS induced HepG2 cells to undergo EMT. YAP is known to play an essential role in cell mechanotransduction and the onset of multiple diseases [15]. Interestingly, we found that YAP was highly expressed in primary human HCC biopsies (Fig. 3A) and HepG2 cells (Fig. 3B), implying that YAP may contribute to the regulation of human HCC development. Immunostaining showed that YAP was mainly localized on the cell membrane of HCC cells (Fig. 3C). The activation of YAP is known to be controlled by its nuclear shuttling and reduced expression of p‐YAP in the cytoplasm [37]. In the FSS‐HepG2 cells, YAP was significantly elevated at both the protein and mRNA levels (Fig. 3D). Moreover, the expression levels of nuclear YAP in FSS‐HepG2 cells were positively correlated with their exposure duration to FSS (Fig. 3E, upper panel), while the cytoplasmic p‐YAP was decreased over time (Fig. 3E, lower panel). YAP was found to be highly expressed in another two HCC cell lines after subjecting to FSS (Fig. 3F). The nuclear translocation of YAP and decreased p‐YAP induced by FSS were also confirmed by western blotting in FSS‐HepG2 cells (Fig. 3G,H). Furthermore, the mRNA levels of the YAP/TAZ target genes (*CTGF* and *AREG*) were also elevated in the FSS‐HepG2 cells (Fig. 3I,J). In the mice with either intraperitoneal or orthotopic injection of FSS‐HepG2 cells, we observed elevated mRNA levels of *YAP* gene (Fig. 3K,L) and nuclear accumulation of YAP protein (Fig. 3M,N). These data collectively suggested that YAP can sense and respond to FSS as a mechanotransducer in HCC cells.

### 3.3 YAP is required for the initiation of FSS‐induced EMT in HCC

**Fig. 4:**
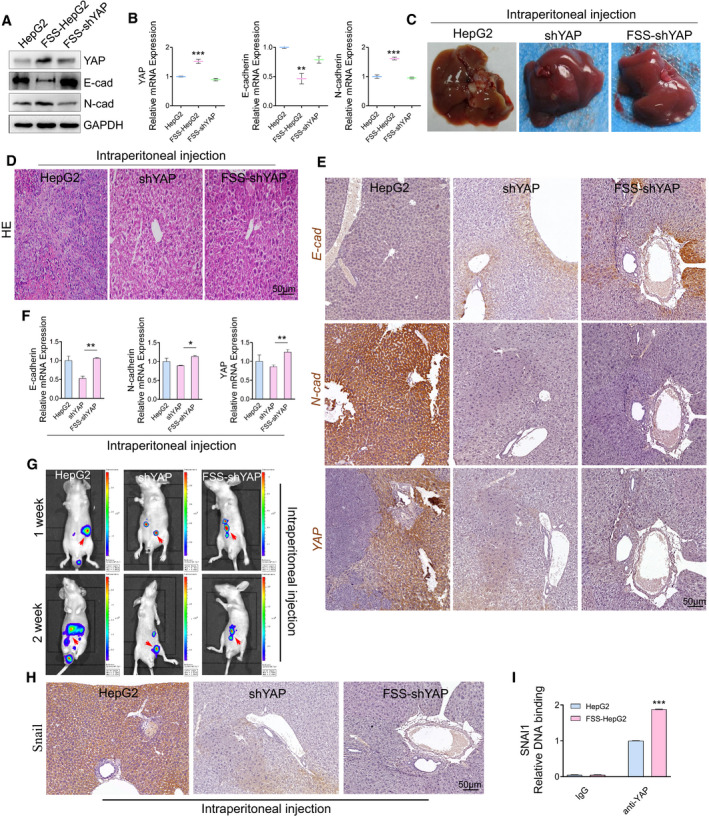
Activation of YAP contributes to EMT induced by FSS. (A) Western blotting analysis of YAP, E‐cad and N‐cad in the indicated HepG2 cells. GAPDH was used as loading control. (B) qPCR analysis of *YAP, E‐cad, N‐cad* in the indicated HepG2 cells (*n* = 3). (C) Representative images of the liver tissues from nude mice injected intraperitoneally with static HepG2, shYAP, or FSS‐shYAP HepG2 cells. (D) HE staining of the liver tumor slices from nude mice injected intraperitoneally with the indicated HepG2 cells. Scale bar: 50 μm. (E) Immunohistochemical staining of EMT genes *E‐cad, N‐cad and YAP* in the liver tumor slices from nude mice injected intraperitoneally with the indicated HepG2 cells (*n* = 3). Scale bar: 50 μm. (F) qPCR analysis of genes involved in EMT. (G) Luciferase live imaging of nude mice injected intraperitoneally with static HepG2, shYAP‐HepG2 or FSS‐shYAP‐HepG2 cells stably expressing luciferase. The arrows indicate the tumor metastasis. (H) Immunohistochemical staining of SNAI1 in liver tumor slices from mice injected intraperitoneally with the indicated HepG2 cells (*n* = 3). Scale bar: 50 μm. (I) ChIP‐qPCR analysis of the association of *YAP* with the promoter of *SNAI1* in HepG2 and FSS‐HepG2 cells. Data are shown as mean ± SEM. Statistics was performed by one‐way analysis of variance followed by Tukey test. *P < 0.05, **P < 0.01, ***P < 0.001.

To verify the role of YAP in EMT induced by FSS in HCC cells, we knocked down YAP by shRNA approach and examined the expression of EMT markers. Knockdown of YAP abolished these mesenchymal transitions indicated by downregulation of E‐cad and up‐regulation of N‐cad in FSS‐HepG2 cells (Fig. 4A,B). We then conducted *in vivo* transplantation for FSS‐HepG2 control and FSS‐shYAP HepG2 cells. Tumorigenesis was significantly dampened in FSS‐shYAP HepG2 cells (Fig. 4C and Fig. S4A). Moreover, HE staining and immunohistochemical staining showed that the malignancy of FSS‐HepG2 cells was significantly decreased by knockdown of YAP (Fig. 4D, Fig. S4B). The mesenchymal architecture in the liver specimens from mice injected with FSS‐shYAP HepG2 cell was markedly abolished (Fig. 4E, Fig. S4C). Enhanced *E‐cad* and reduced *N‐cad* mRNA levels were detected in the liver specimens from mice injected with FSS‐shYAP HepG2 cells (Fig. 4F, Fig. S4D). Live imaging of nude mice showed that the metastasis of FSS‐shYAP HepG2 cells was significantly decreased (Fig. 4G, Fig. S4E). The expression of SNAI1 was decreased in the liver specimens from mice injected with FSS‐shYAP HepG2 cells (Fig. 4F, Fig. S4F). ChIP‐PCR results provided direct evidence that YAP was strongly bound to the promoter of *SNAI1* gene (Fig. 4I). Moreover, the binding of YAP to several well‐characterized YAP target genes was also validated by ChIP‐PCR, suggesting YAP actively regulated the expression of its target genes and promoted EMT upon FSS treatment (Fig. S4G).

We went on to test whether nuclear activation of YAP contributed to the increased motility of HepG2 induced by FSS. We found that the knockdown of YAP indeed repressed the migration and invasion ability of HepG2 cells (Fig. S5A–D). Moreover, the expression of RAC1, RHOA, and CDC42 in the cell membrane was significantly decreased in the shYAP HepG2 (Fig. S5E–I). YAP knockdown inhibited the activation of RAC1 (Fig. S5J) and the phosphorylation of RAC1 and RHOA (Fig. S5K). Besides, the expression of metallomatrix protease (MMP‐3) was significantly down‐regulated in the shYAP HepG2 cells (Fig. S5L). Most importantly, knockdown of YAP in FSS‐HepG2 cells (FSS‐shYAP HepG2) abolished the increased motility and induced expression of RAC1, RHOA, and CDC42 induced by FSS (Fig. S6A–D).

A recent study reported that YAP bound to the enhancers of 379 target genes and directly regulated their expression [8]. We focused on these YAP target genes in our global gene profiling data. Among the 379 genes, we found that 34 and 66 genes were up‐regulated and down‐regulated in the FSS HepG2 cells, respectively (Table S6). Moreover, KEGG analysis indicated that the YAP target genes identified in our gene profiling analysis are involved in FSS and a few other signaling pathways (*P* < 2.98 × 10^−3^; Fig. S6E). Heatmap of the top 25 differently expressed YAP target genes was shown in Fig. S6F. Eight YAP target genes involved in cell motility and identified in our gene profiling analysis were further verified by qPCR (Fig. S7, Table S7). ChIP‐PCR analyses showed that YAP bound to the promoter of Rho GTPase *CDC42, RHOA and RAC1* with various affinities (Fig. S6G–I). The above data suggest that YAP‐mediated transcription plays an active and critical role in FSS induced HCC EMT and cell motility.

### 3.4 Integrins regulate the mechanotransduction of YAP

**Fig. 5:**
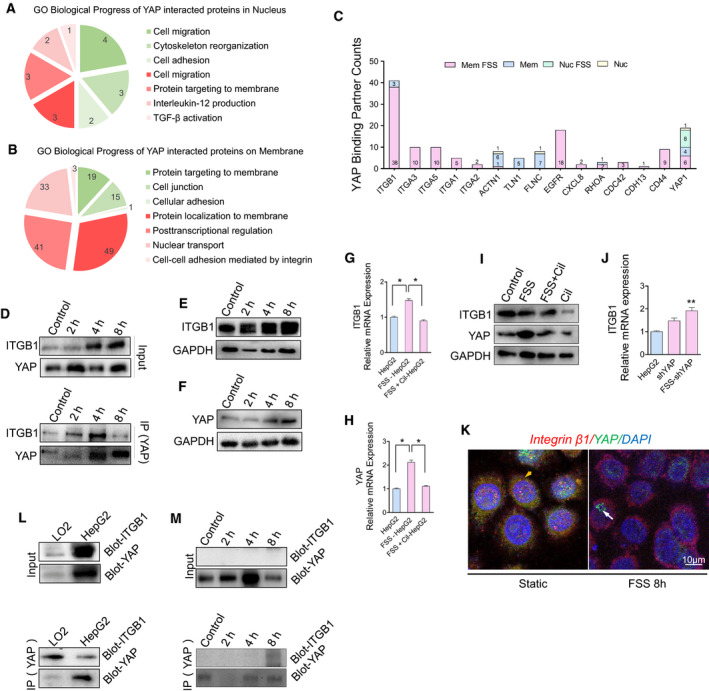
FSS triggers the release of YAP from integrin and its translocation to the nucleus. (A,B) BP analysis on the proteins identified in the label‐free quantitative proteomics analysis for YAP‐interacting proteins in the plasma membrane and nuclear fractions. (upregulation colored red, downregulation colored green). (C) Counts in the label‐free quantitative proteomics analysis for a panel of selected binding partners of YAP in response to FSS. (D) Co‐IP analysis of YAP and ITGB1 interaction in the membrane fraction of static cultured HepG2 and FSS‐HepG2 cells. (E,F) Western blotting analysis of YAP and ITGB1 in HepG2 cells treated with FSS for the indicated time durations. (G–I) The mRNA and protein levels of ITGB1 and YAP in HepG2, FSS‐HepG2 and FSS + Cil‐HepG2 cells treated with the integrin inhibitor cilengitide (*n* = 3). (J) FSS induces the mRNA level of integrin in cells with YAP knockdown (*n* = 3). (K) Immunostaining showing the colocalization of integrin and YAP under static and FSS conditions. The arrows indicated the location of YAP. (L) Co‐IP analysis of YAP and ITGB1 interaction in the membrane fraction of LO2 and HepG2 cells at the static state. (M) Co‐IP analysis of YAP and ITGB1 interaction in the nuclear fraction of static cultured HepG2 and FSS‐HepG2 cells. Data are shown as mean ± SEM (*n* = 3, by one‐way analysis of variance followed by Tukey test, *P < 0.05, **P < 0.01).

To gain insights into how YAP is activated by FSS, which in turn promotes malignant EMT status and HCC cell motility, we conducted a label‐free quantitative proteomics analysis to identify the interacting protein partners of YAP. FSS‐HepG2 cells were separated into nuclear and membrane fractions. Both fractions were subjected to Co‐IP using an established YAP antibody. The potential interacting partners were subsequently analyzed by MS. BP enrichment analysis showed that protein candidates in the nuclear fraction are associated with the BP term related to cell migration and cytoskeleton rearrangement which is consistent with the important function of nuclear YAP in the cells treated with FSS (Fig. 5A). Notably, in the membrane fraction, FSS induced the activation of the integrin‐related signal pathway and initiation of nuclear transport (Fig. 5B). Importantly, multiple integrin family proteins were identified as binding partners of YAP in the membrane fraction (Fig. 5C). Integrin β subunits play essential roles in the mechanotransduction of hemodynamic forces to biochemical signals. Among them, integrin β1 (ITGB1) is a direct sensor of unidirectional flow for keeping vascular homeostasis [38]. Co‐precipitation of YAP and ITGB1 in the membrane fraction of FSS‐HepG2 cells was further confirmed by western blotting on the Co‐IP samples (Fig. 5D). Moreover, the expression of ITGB1 and YAP was induced with a similar trend according to FSS tendency (Fig. 5E,F). Cilengitide, an inhibitor of ITGB1 [39], suppressed the FSS‐induced increased expression of ITGB1 and YAP (Fig. 5G–I). Knockdown of YAP did not suppress the increase of ITGB1 expression (Fig. 5J), suggesting that YAP did not mediate ITGB1 expression induced by FSS. YAP was induced to dissociate from ITGB1 at the membrane in HepG2 cells after FSS treatment (Fig. 5K), but the interaction of YAP and ITGB1 was not detected in the nuclear fraction under any conditions (Fig. 5M). Notably, the co‐precipitation of ITGB1 with YAP was also detected in static LO2 (Fig. 5L). Collectively, these data revealed that YAP was detached from its binding partner ITGB1 in the cell membrane to facilitate its nuclear translocation.

### 3.5 F‐actin mediates the mechanotransduction of YAP through GEF‐H1

**Fig. 6:**
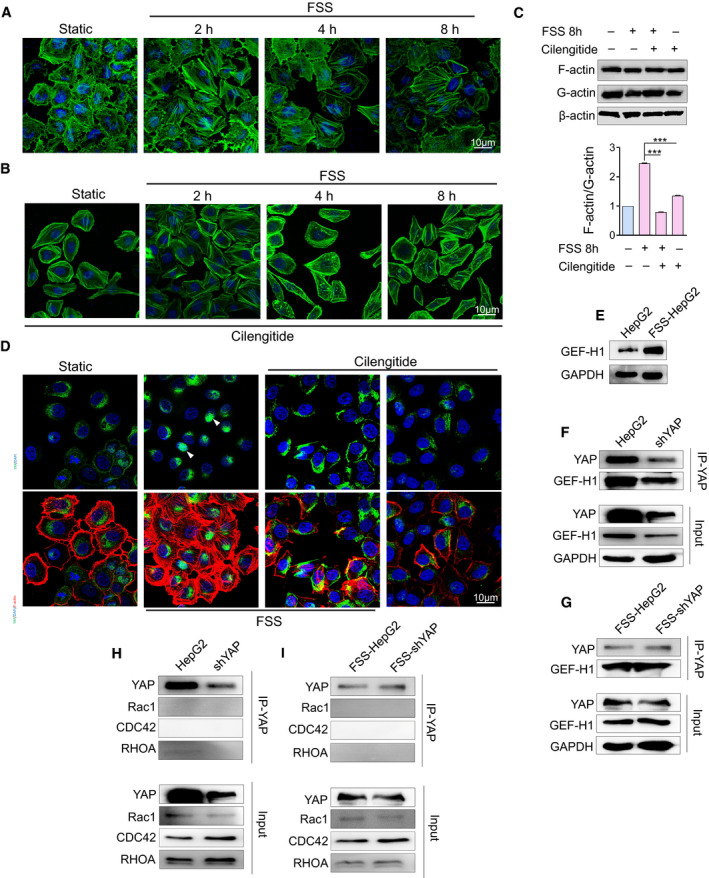
F‐actin transmits the biomechanical signal from integrin to YAP through GEF‐H1. (A) Cytoskeleton arrangement induced by FSS was examined using BODIPY (green stain) at the indicated time points after FSS treatment (*n* = 3). Scale bar: 10 μm. (B) Inhibition of the mechanotransduction of integrin induces the disruption of F‐actin (*n* = 3). Scale bar: 10 μm. (C) Western blotting analysis of the G‐actin/F‐actin ratio in HepG2, FSS‐HepG2, FSS+Cil‐HepG2 and Cil‐HepG2 cells (*n* = 3). Statistical analyses was performed by one‐way analysis of variance followed by Tukey test. Data are shown as mean ± SEM. ***P < 0.001. (D) Cilengitide induces the disruption of F‐actin (red) and reduces YAP (green) nuclear accumulation (*n* = 3). The arrows indicate the location of YAP. Scale bar: 10 μm. (E) Western blotting analysis of GEF‐H1 in HepG2 and FSS‐HepG2 cells. (F–I) Co‐IP analysis of control and YAP‐knockdown HepG2 cells subjected to FSS (*n* = 3).

ITGB1 is an important molecular player in focal adhesions (FAs) and is known to bind to F‐actin through adaptor proteins, including Talin. It is plausible to speculate that ITGB1 may function as a biomechanical signal transducer for F‐actin and regulate cell motility [40]. Actin was co‐precipitated with YAP in the membrane fraction (Fig. 5C). We therefore decided to explore the role of actin in YAP activation. We found that FSS induced remodeling and rearrangement of F‐actin fiber (Fig. 6A). Inhibition of integrins by cilengitide dramatically disrupted F‐actin fiber in both static and FSS‐HepG2 cells (Fig. 6B). Western blot analysis of the ratio between F‐actin and G‐actin indicated impairment of actin bundle polymerization in FSS‐HepG2 cells treated with cilengitide (Fig. 6C). Moreover, the nuclear accumulation of YAP was attenuated in FSS‐HepG2 cells treated with cilengitide (Fig. 6D), suggesting that inhibition of integrins may impede the mechanical signal transduction of YAP through disrupting F‐actin fiber bundles.

It has been reported that the actin‐bound GEF responds acutely to integrin β‐mediated extracellular mechanical cues [41]. We found that FSS triggered the increase of GEF‐H1 expression and fiber polymerization F‐actin in a similar way (Fig. 6E). Interestingly, GEF‐H1 physically interacted with YAP in both static HepG2 cells and FSS‐HepG2 cells (Fig. 6F,G). In contrast, the well‐known GEF target proteins Rho GTPases (RAC1, RHOA and CDC42) did not exhibit physical interaction with YAP (Fig. 6H,I). These data suggest that GEF‐H1 contributes to the nuclear activation of YAP by transmitting the biomechanical signal from F‐actin.

### 3.6 YAP modifies the mRNA methylation of EMT‐related genes

**Fig. 7:**
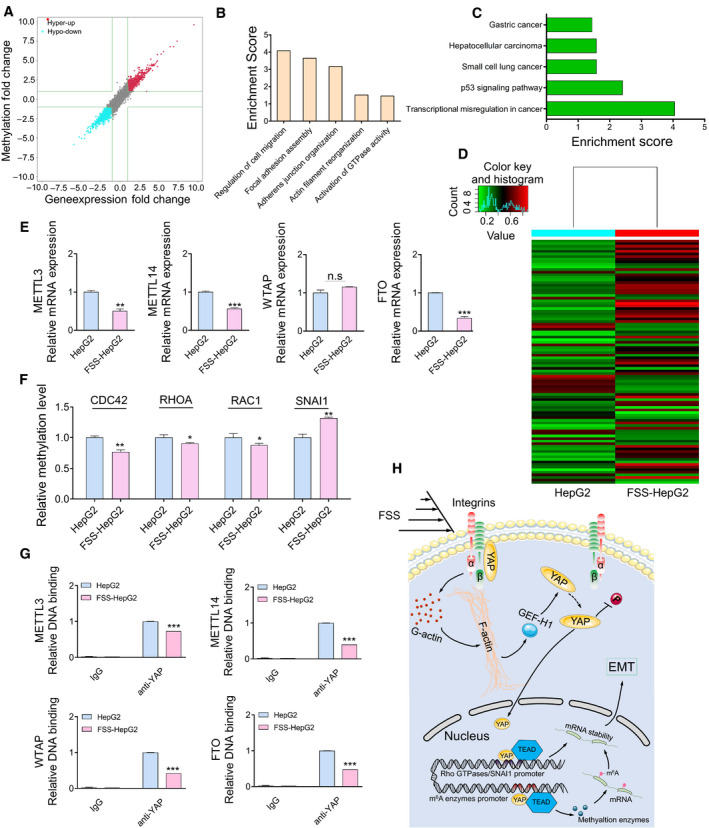
YAP modulates the m^6^A RNA methylation of EMT ‐related genes. (A) The distribution of transcript with a significant change in both m^6^A level and expression in the comparison of FSS‐HepG2 with HepG2 cells. (B) Major enriched and meaningful BP terms of m^6^A peaks transcripts. (C) Significantly enriched pathways of m^6^A peak transcripts. (D) Hierarchical clustering analysis of the differentially methylated mRNA. (E) qPCR analysis of *METTL3, METTL14, WTAP* and *FTO* in HepG2 and FSS‐HepG2 cells. (F) The methylation level analysis of EMT‐related genes identified in m^6^A‐mRNA microarray data. (G) ChIP‐qPCR showing fold changes of the association of YAP with the promoter of *METTL3, METTL14, WTAP* and *FTO*. (H) FSS induces YAP separation from integrin β subunits. Integrin β subunits transmit the biomechanical signal to F‐actin, which results in translocation of YAP to the nucleus through GEF‐H1. The activated nuclear YAP triggers EMT through transcriptional and post‐transcriptional modification of EMT‐related genes. Data are shown as mean ± SEM (*n* = 3, by two‐tailed unpaired *t*‐test, *P < 0.05, **P < 0.01, ***P < 0.001, n.s. denoting no significance).

RNA can undergo epitranscriptomic changes, which lead to post‐transcriptional regulation of gene expression. One of the most prevalent internal modifications of mRNA is m^6^A, which has emerged as a widespread regulatory mechanism for gene expression [42]. Microarray profiling showed that 1062 and 2388 transcripts were significantly hypermethylated and hypomethylated, respectively. Interestingly, the methylation status of genes showed positive correlation with their expression levels in FSS‐HepG2 cells (Fig. 7A). GO and KEGG pathway analyses indicated that methylation is associated with cell migration activity and HCC (Fig. 7B,C). Hierarchical Clustering revealed distinguishable gene expression patterns between HepG2 and FSS‐HepG2 cells (Fig. 7D). The qPCR analyses of *METTL3*, *METTL14*, *WTAP*, and *FTO*, which regulate most of m^6^A methylation events of mRNA [43], suggested a reduced expression of *METTL3*, *METTL14* and *FTO* in FSS‐HepG2 cells in comparison with static HepG2 cells (Fig. 7E). Moreover, we found that *SNAI1* mRNA showed significantly increased methylation (Fig. 7F), which might contribute to the increased expression of *SNAI1* mRNA induced by FSS (Fig. 1C). We next exploited the involvement of YAP in the global methylation levels of mRNA in HepG2 cells. ChIP‐PCR provided direct evidence showing that YAP bound to *METTL3, METTL14, WTAP* and *FTO* (Fig. 7G) in static HepG2 cells. Moreover, FSS appeared to dampen the binding of YAP to the promoter of these genes. Nevertheless, these data implied that YAP regulated the m^6^A modification of EMT‐related genes through controlling the transcription of multiple m^6^A modulators. Taken together, activated YAP in the nucleus could regulate the expression of EMT‐related genes by either initiating transcription or modifying methylation to promote high EMT status in FSS‐HepG2 cells.

## 4 Discussion

Hepatocellular carcinoma accounts for approximately 90% of all primary liver cancer cases [44]. High metastasis and invasion is a hallmark of human HCC and the leading cause of poor prognosis. Therefore, an improved understanding of the molecular mechanism behind HCC cell motility is essential for the identification of potential targets for diagnostic and therapeutic intervention. EMT, characterized by the loss of apicobasal polarity and intercellular adhesion, and acquisition of mesenchymal cell morphology and motility by cytoskeleton remodeling, is crucial for cancer invasion and metastasis [2]. EMT has been shown to trigger the dissociation of cancer cells from primary carcinomas and promote their subsequent migration and dissemination to distant sites [45]. Biochemical cues, such as inflammatory chemokine and cytokines, growth factors and extracellular matrix components play essential roles in the activation of EMT. On the other hand, biophysical cues such as FSS can also induce EMT process of tumor cells and promote their survival in hematogenous dissemination [46, 47]. Notably, the FSS in the tumor environment is significantly different than that in vessels. Due to the rich blood microvasculature in the liver, HCC should be considered a tumor type that initiates and develops in a unique microenvironment. The FSS exposure of HCC cells in the liver is approximately 0.1–2 dyn·cm^−2^ [6]. Until now, however, the effect of FSS in the HCC microenvironment on regulating tumor cell EMT is still not well studied.

Most previous studies about the impact of FSS on modulating cancer cell motility were conducted *in vitro*. In the present study, we executed intraperitoneal and orthotopic tumor transplantation with HCC cells subjected to FSS. Liver tissues from mice injected with FSS‐HepG2 cells showed increasing tumorigenesis and invasion (Fig. 1J–M). FSS‐pretreated HepG2 cells spread faster than control HepG2 cells in the recipient mice (Fig. 1H,I). To further illustrate whether the increased cell motility induced by FSS is associated with EMT, we checked the EMT status both *in vitro* and *in vivo*. SNAI1, a key EMT inducer, showed higher expression in the HCC cell line HepG2 cells than in the non‐transformed LO2 cells. Its expression was further induced by FSS in HepG2 cells (Fig. 1B). ChIP‐PCR results showed YAP acted on the promoter region of *SNAI1* to promote its expression induced by FSS. These results suggest that YAP is able to drive the expression of *SNAI1* directly, promoting the activation of EMT when HCC cells are exposed to FSS.

YAP is a key mediator of the Hippo pathway [48]. Mechanical cues, such as stiffness, cell contact, cell geometry, and cell attachment status regulate the Hippo pathway by modulating the activity of Rho GTPases, which in turn affects the activation of YAP. Therefore, YAP is regarded as a downstream factor of Rho GTPases. However, activation of YAP by mechanical cues can be independent of the Hippo pathway. For example, it was shown that mechanical strain mediated nuclear accumulation and transcriptional activity of YAP through E‐cad extracellular engagement [49]. The SWI/SNF complex was also found to serve as a mechano‐regulated inhibitor of YAP and TAZ, independent of the Hippo pathway and Rho GTPases [50]. In the present study, we did not detect MST1/2 and LATS1/2 in our label‐free quantitative proteomics analyses of the cells treated with FSS, suggesting that YAP activation by FSS may be independent of the Hippo pathway in our model systems.

Integrin is a well‐known mechanotransducer on cell membrane in response to FSS [51]. However, the biomechanical signal transiting mechanism is far from clear. Here, we identified ITGB1 as a physically binding partner of YAP. Moreover, dissociation of YAP from the protein complex with ITGB1 on the cell membrane promotes its subsequent translocation from the cytoplasm to the nucleus (Fig. 5). Nuclear activation of YAP further triggered EMT through activating the expression of SNAI1 and suppressing the expression of m^6^A modulators (Figs S4 and S5).

## 5 Conclusion

We demonstrated that the FSS induces EMT in HCC cells and promotes their invasion and metastasis. YAP was a key mediator for the activation of EMT and enhanced cell mobility induced by FSS. ITGB1 was identified as a membrane binding partner of YAP, and the release of YAP from ITGB1 to the cytosol was found to be an initial step in the nuclear translocation of YAP. Moreover, after sensing the biomechanical signal, the integrin β subunit initiates F‐actin polymerization and induces high expression of GEF‐H1, efficiently facilitating the translocation of YAP to nuclei, whereby YAP executes its function as a TF to regulate the expression of EMT‐related genes (Fig. 7H).

## Conflict of interest

The authors declare no conflict of interest.

## Author contributions

HY carried out all the experimental work and drafted the manuscript. JH and YueW directed the animal study and provided Human primary tumor samples. JH and GS participated in western blotting, pull‐down and Rac1 activity assay. YS and FF participated in immunohistochemical, immunofluorescence staining and cytoskeletal F‐actin staining, and performed the confocal microscopy analyses. KG improved the parallel plate chamber, worked on the numerical simulation, and performed the bioinformatics and statistical analysis. WY and NYF revised the manuscript. YunBW, YS and XL led the project, designed the experiments and edited the manuscript. All authors read and approved the final manuscript.

### Peer Review

The peer review history for this article is available at https://publons.com/publon/10.1002/1878‐0261.13061.

## Supporting information


**Fig. S1**. The motility of HepG2 cells is associated with EMT.
**Fig. S2**. The numerical simulation of parallel plate chamber.
**Fig. S3**. FSS induces EMT in HCC cells.
**Fig. S4**. Nuclear activation of YAP accelerates the EMT of HepG2 *in vivo*.
**Fig. S5**. YAP modulates the expression of Rho GTPase in HepG2 cells.
**Fig. S6**. The transcriptional function of YAP modulates cell motility.
**Fig. S7**. FSS elevates the expression of YAP‐targeted migration genes.
**Table S1**. Detailed information of antibodies.
**Table S2**. PCR primers used for quantitative ChIP‐PCR in this study.
**Table S3**. PCR primers used for quantitative RT‐PCR in this study.
**Table S4**. Silencing YAP target sequence.
**Table S5**. Gene lists involved in migration.
**Table S6**. YAP positive target genes from HepG2 VS FSS‐HepG2.
**Table S7**. YAP‐targeted genes involved in migration.

## Data Availability

Gene and protein expression profiling data and methylation microarray data have been deposited for public access in the NCBI Gene Expression Omnibus under Accession numbers GSE146169 and GSE146090. Other data and materials used for this study are available from the corresponding author on request.
