# Supplementary material for: Fluid shear stress activates YAP to promote epithelial–mesenchymal transition in hepatocellular carcinoma
Source: Mol Oncol. 2021 Aug 2;15(11):3164–83. doi: 10.1002/1878-0261.13061 (PMC8564657; doi:10.1002/1878-0261.13061)
Supplement: Supplementary file 1 — Fig. S1. The motility of HepG2 cells is associated with EMT. Fig. S2. The numerical simulation of parallel plate chamber. Fig. S3. FSS induces EMT in HCC cells. Fig. S4. Nuclear activation of YAP accelerates the EMT of HepG2 in vivo. Fig. S5. YAP modulates the expression of Rho GTPase in HepG2 cells. Fig. S6. The transcriptional function of YAP modulates cell motility. Fig. S7. FSS elevates the expression of YAP‐targeted migration genes. Table S1. Detailed information of antibodies. Table S2. PCR primers used for quantitative ChIP‐PCR in this study. Table S3. PCR primers used for quantitative RT‐PCR in this study. Table S4. Silencing YAP target sequence. Table S5. Gene lists involved in migration. Table S6. YAP positive target genes from HepG2 VS FSS‐HepG2. Table S7. YAP‐targeted genes involved in migration. [file MOL2-15-3164-s001.docx]

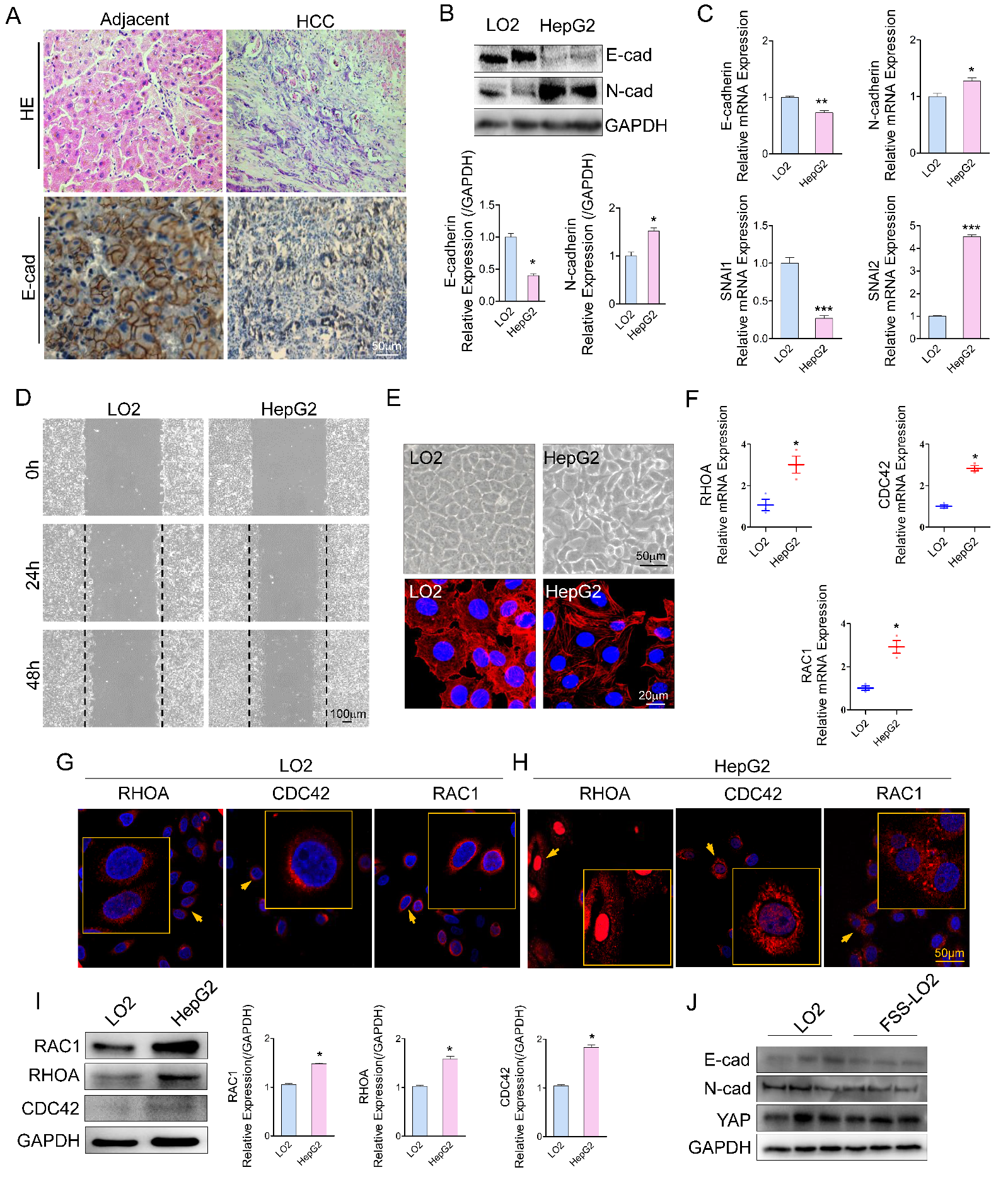
**Fig. S1 The motility of HepG2 cells is associated with EMT.** (**A**) Immunohistochemical staining of the epithelial marker gene (E-cad) in the liver tumor of representative human HCC specimens (n=3). (**B**) Western blotting analysis of proteins (E-cad and N-cad) involved in EMT. GAPDH was used as the internal control; the quantification of grey value was analyzed by Image J (n=3). (**C**) qPCR analysis of genes involved in EMT (n=3). (**D**) Wound healing assay shows the difference in cell motility between LO2 and HepG2 cells. Scale bar, 100 μm. (**E**) Cytoskeleton stained with Phalloidine. Scale bar, 20 μm. (**F**) qPCR analysis of genes involved in migration (n=3). (**G, H**) Immunostaining of Rho GTPases in LO2 and HepG2 cells. Scale bar, 50μm. (**I**) Western blot analysis of Rho GTPases in LO2 and HepG2 cells. (**J**) Western blot analysis of EMT-TFs and YAP in LO2 and LO2 subjected with FSS. GAPDH was used as internal control. Quantification of grey value is analyzed by Image J (n=3). Data are presented as mean ± s.e.m; statistics are performed by a two-tailed unpaired t-test, ^*^*P*＜0.05.


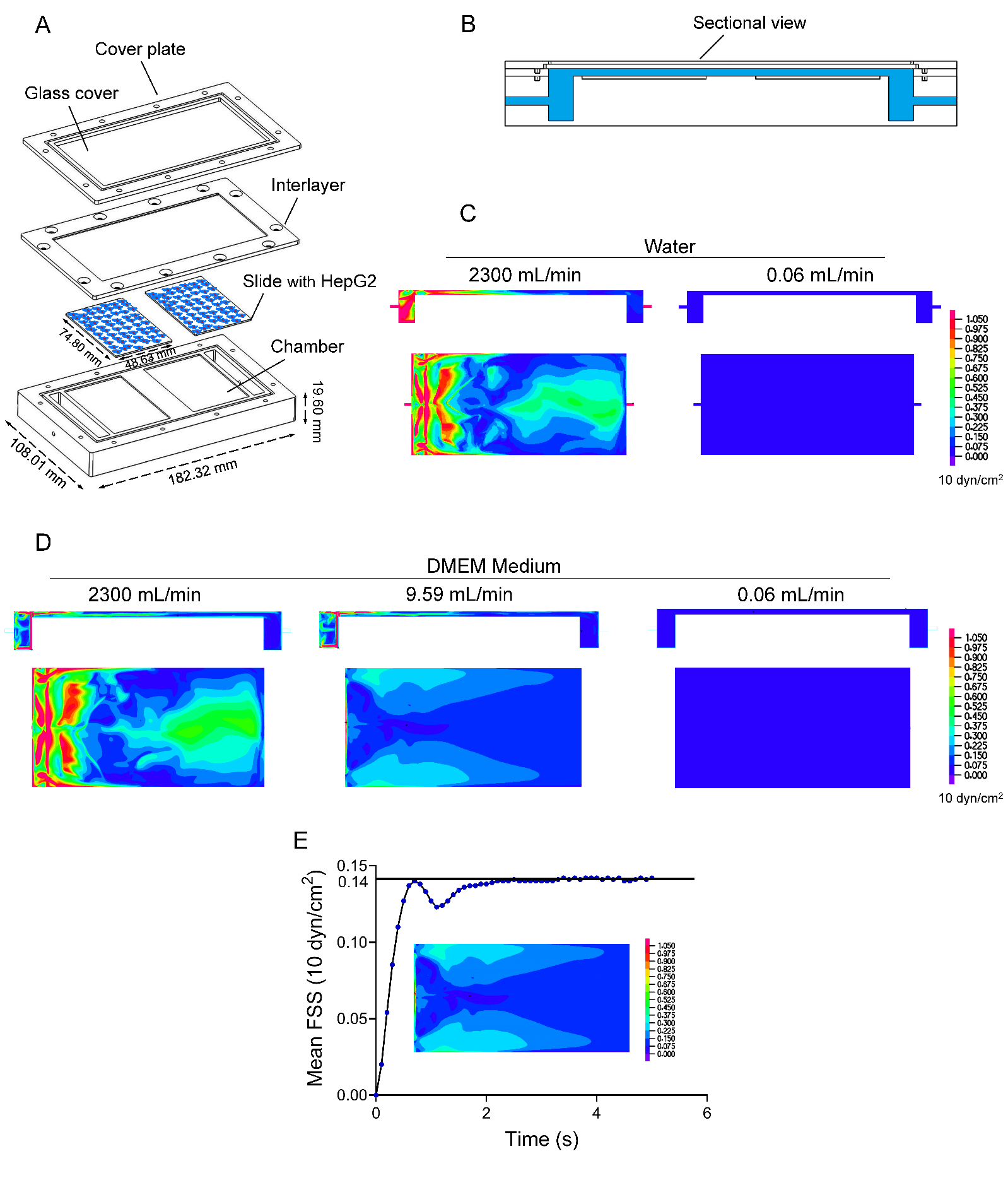
**Fig. S2 The numerical simulation of parallel plate chamber.** (**A**) The schematic diagram of the parallel plate chamber and the profile map of the parallel plate chamber. The blue region is the pathway of fluid. (**B**) The sectional view of the modified chamber. (**C**) The fluid shear stress in the different areas of the chamber; the fluid is water. (**D**) The fluid shear stress in the different areas of the chamber; the fluid is DMEM medium. 2300 mL/min is the maximum velocity of M_ASTER_FL_EX_^®^L/S^®^ (Modle 77200-50). 0.06 mL/min is the minimum velocity of M_ASTER_FL_EX_^®^L/S^®^ (Modle 77200-50). Re < 2000 contact with the zone of the cell. (**E**) The mean FSS analysis in the chamber when the fluid volume is 9.59 mL/min.


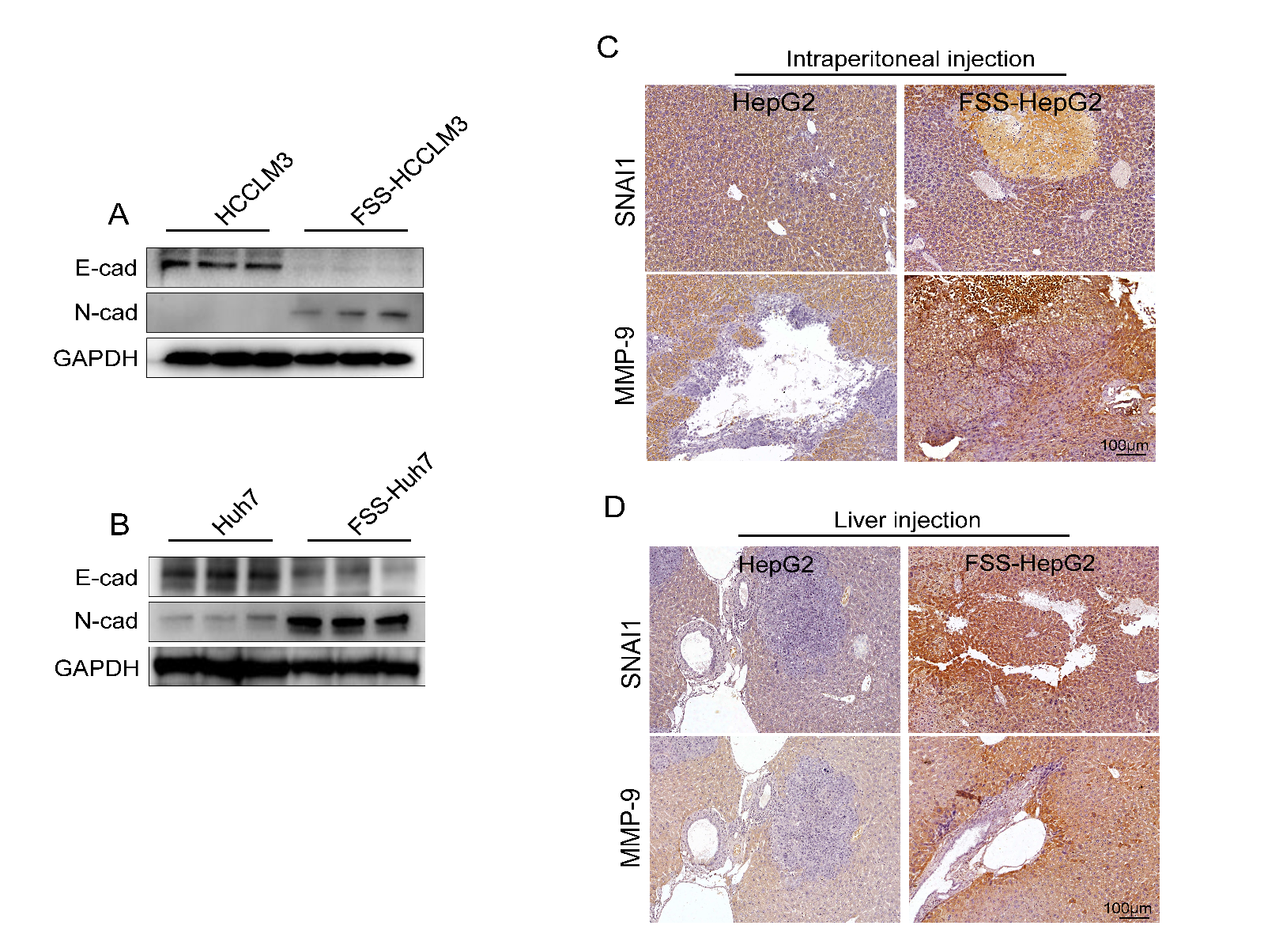
**Fig. S3 FSS induces EMT in HCCs cells. (A, B)** Western blotting analysis of EMT markers in HCC cells and FSS-HCC cells. GAPDH is used as internal control (n=3). **(C, D)** Representative images showing immunohistochemical staining of Snai1 and MMP-9 in the liver tissue from the mice intraperitoneally and orthotopically injected with static cultured HepG2 or FSS-HepG2 cells. Scale bar, 100 μm (n=3).


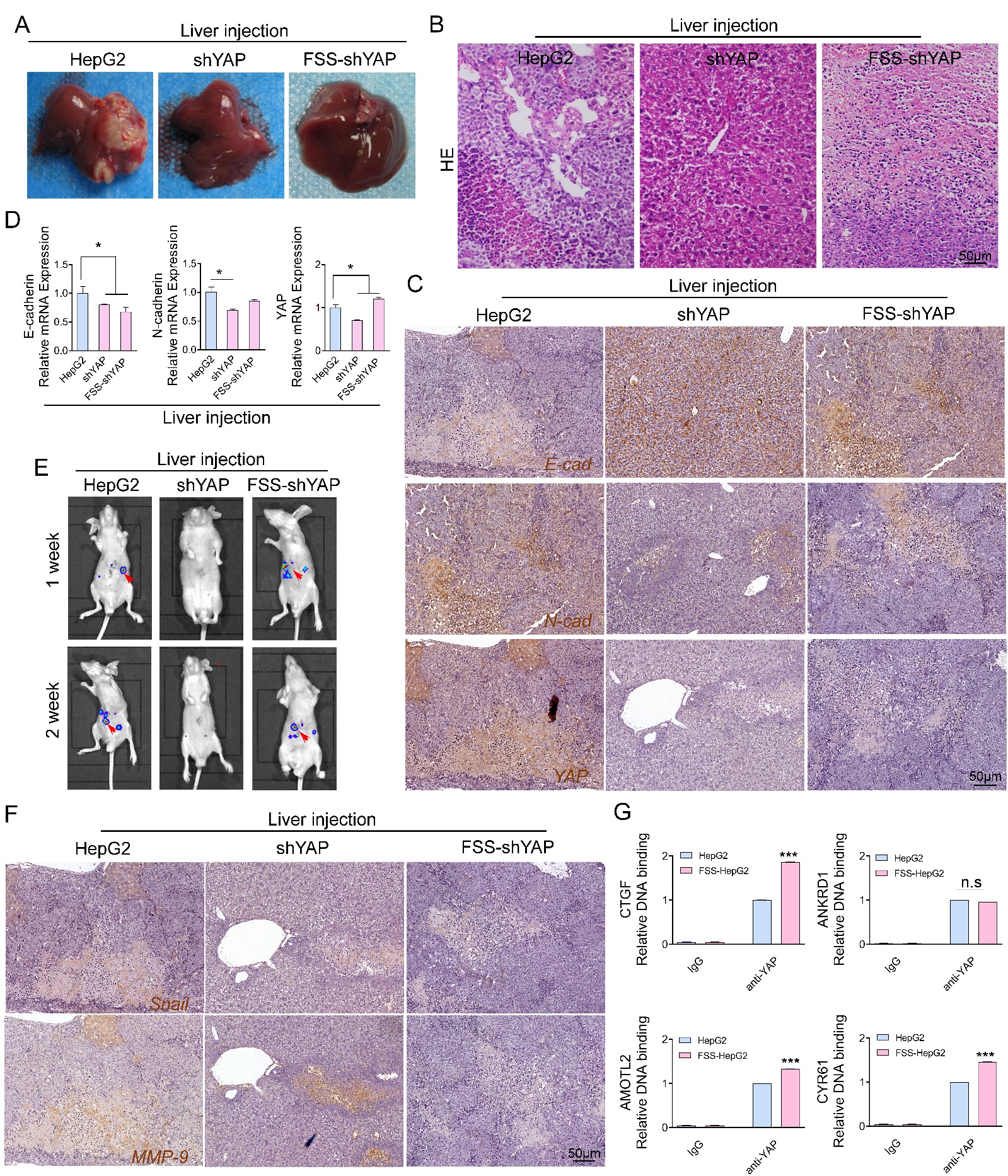
 **Fig. S4 Nuclear activation of YAP accelerates the EMT of HepG2 *in vivo.*** (**A**) Representative images of liver tissues obtained from the nude mice with orthotopical injection of static HepG2, shYAP, or FSS-shYAP cells, respectively. (**B**) HE staining of liver tumor slices from the mice with orthotopical injection. Scale bar, 50 μm. (**C**) Immunohistochemical staining of EMT genes using liver tumor slices from the mice with orthotopical injection (n=3). Scale bar, 50 μm. (**D**) qPCR analysis of genes involved in EMT. (**E**) Luciferase live imaging of the nude mice with orthotopical injection of static HepG2, shYAP-HepG2, or FSS-shYAP-HepG2 cells. All cells are co-transfected with luciferase. The arrows indicated the tumor metastasis. (**F**) Immunohistochemical staining of Snai1 and MMP-9 using liver tumor slices from mice with orthotopic injection (n=3). Scale bar, 50 μm. (**G**) Validation of YAP binding sites by ChIP-qPCR. Data are shown as mean ± s.e.m. Statistics was done by one-way analysis of variance followed by Tukey test, ^*^*P*＜0.05, ^**^*P*＜0.01, ^***^*P*＜0.001.


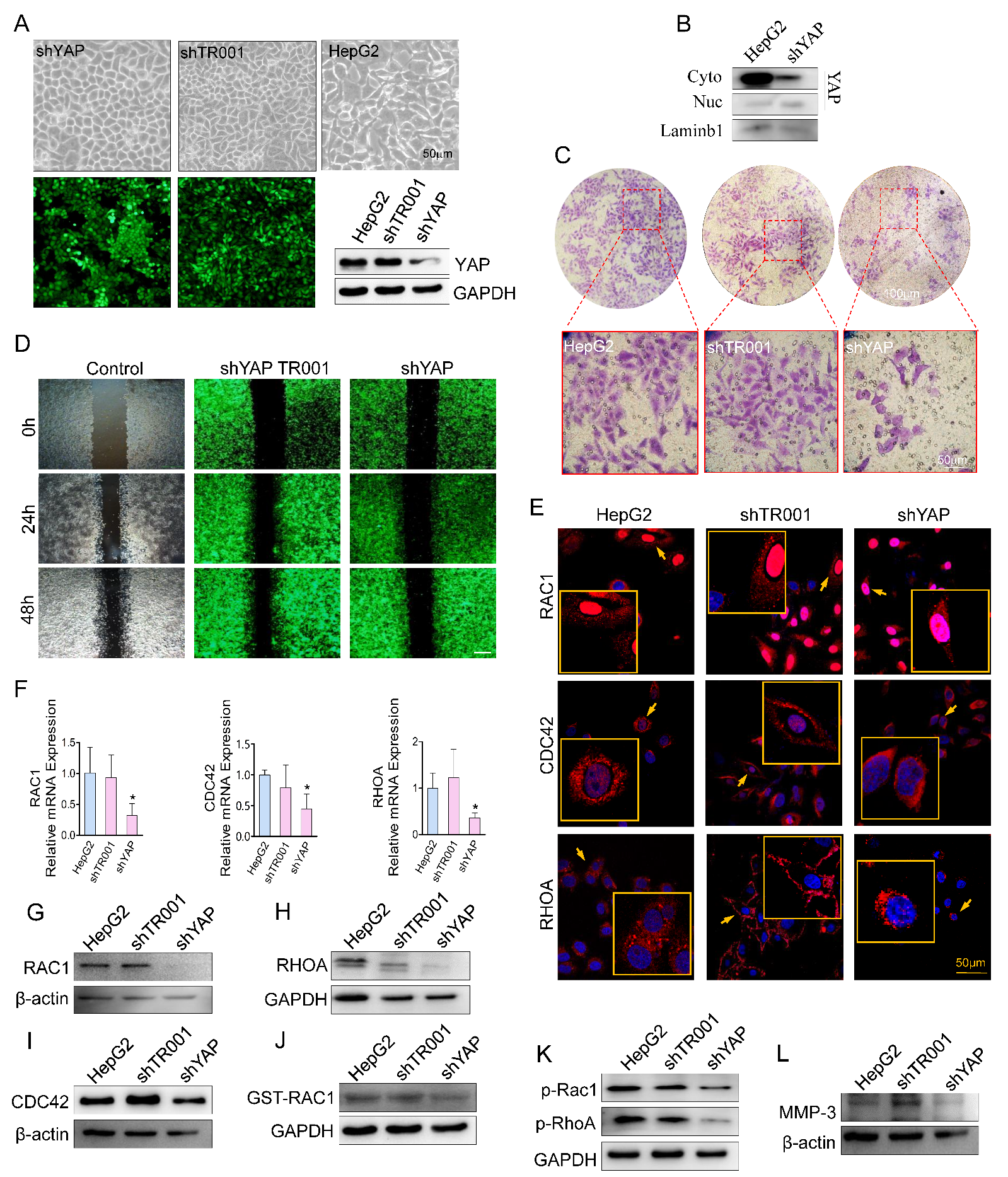
**Fig. S5 YAP modulates the expression of Rho GTPases in HepG2 cells.** (**A**) Validation of transfection efficiency. (**B**) Western blotting analysis of MMP-3 in cytoplasmic and nuclear fractions of shYAP HepG2 cells. (**C**) Measurement of the invasion ability of HepG2 cells in a transwell. Scale bar, 50 μm. (**D**) Wound healing assay reveals the migration ability of HepG2 cells with indicated treatment. Scale bar, 100 μm. (**E**) Immunostaining of Rho GTPases in HepG2 cells with indicated treatment, Scale bar, 50 μm. (**F**) qPCR analysis of genes involved in migration. Data are presented as mean ± s.e.m. Statistics was performed by one-way analysis of variance followed by Tukey test, ^*^*P*＜0.05. n=3. (**G-I**) Western blotting analysis of Rho GTPases in HepG2 cells with indicated treatment. GAPDH and β-actin are used as internal control (n=3). (**J**) Pull-down assay of activated Rho GTPases. (**K**) Western blotting analysis of activated Rho GTPases. (**L**) Western blotting analysis of MMP-3 in HepG2 cells with indicated treatment.


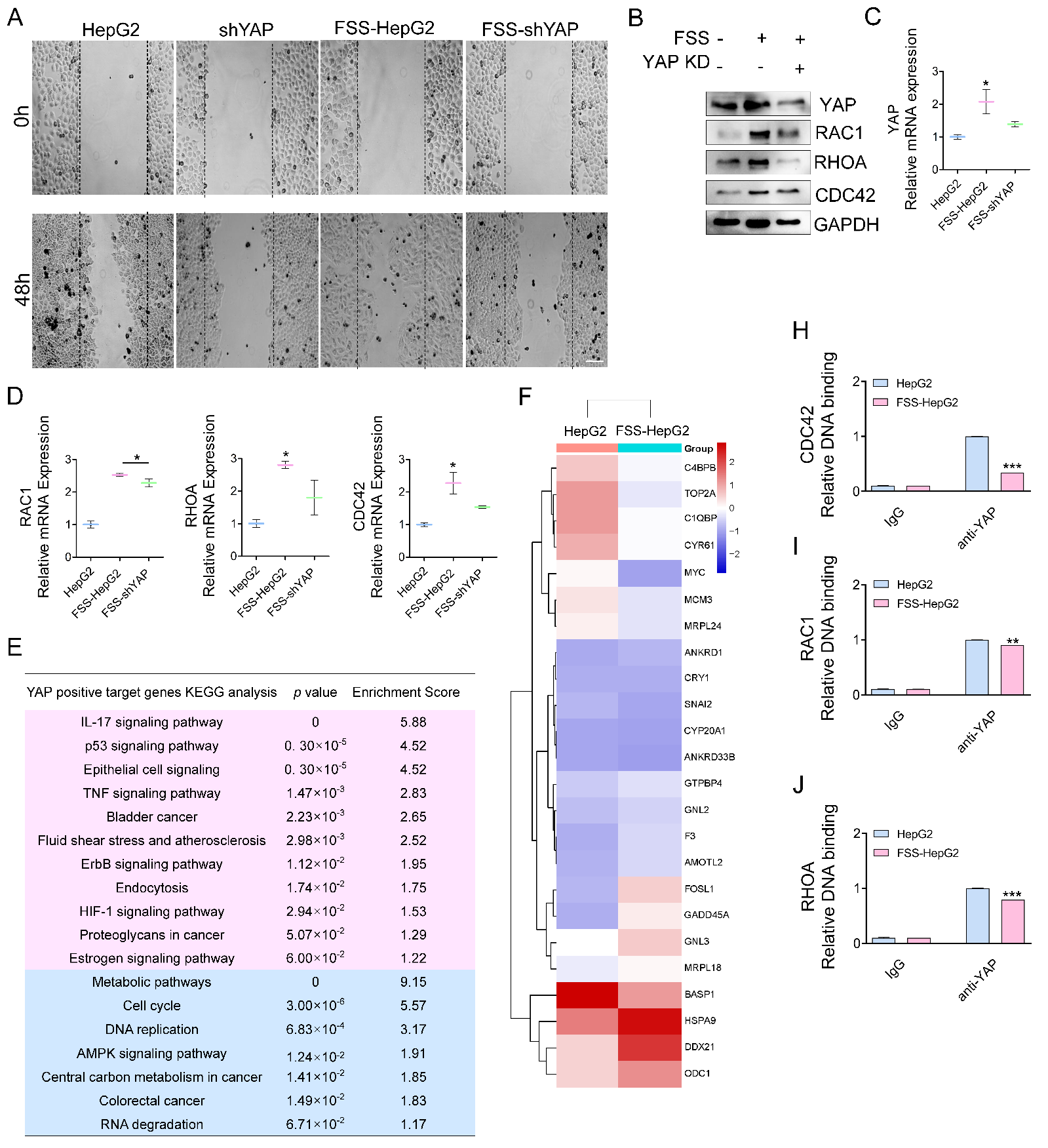
**Fig. S6 The transcriptional function of YAP modulates cell motility.** (**A**) Wound healing assay reveals the migration ability in FSS-shYAP. Scale bar, 50 μm. (**B**) Western blotting analysis of Rho GTPases in HepG2 cells with indicated treatment. GAPDH is used as internal control (n=3). (**C, D**) qPCR analysis of Rho GTPases in HepG2 cells with indicated treatment (n=3). Data are presented as mean ± s.e.m; statistics were performed by one-way analysis of variance followed by Tukey test, ^*^*P*＜0.05. (**E**) KEGG pathway enrichment analysis reveals roles for FSS in a number of signal pathways modulated by YAP target genes. (**F**) Genes cluster regulated by YAP in FSS-HepG2 were evaluated compared with static cells. The color key represents normalized expression within rows. (**G-I**) ChIP-qPCR showing fold changes of YAP binding to the promoter of Rho-GTPases (*CDC42, RHOA,* and *RAC1*) in FSS-HepG2 cells.

**
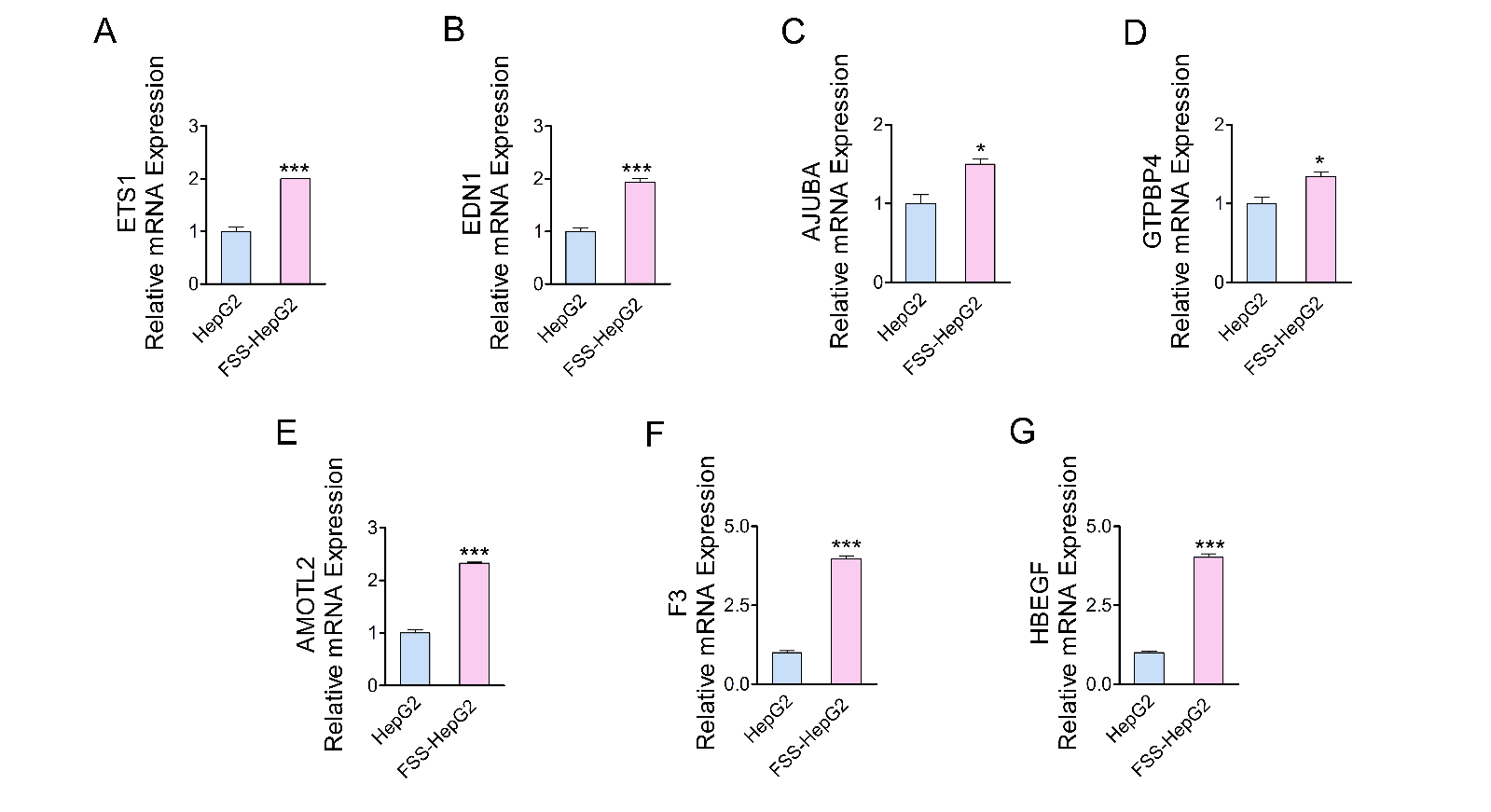
Fig. S7 FSS elevates the expression of YAP-targeted migration genes.** (**A-G**) qPCR analysis of 7 increased YAP-targeted migration genes. Data are presented as mean ± s.e.m; statistics were performed by two-tailed unpaired t-test, ^*^*P*＜0.05, ^***^*P*＜0.001, n=3.

**Table S1** Detailed information of antibodies.

| **Category** | **Antibody** | **Isotype** | **Manufacturer** | **Cat. No** |
| --- | --- | --- | --- | --- |
| **Internal control proteins** | GAPDH | Mouse mAb | Zhongshanjinqiao | TA-08 |
|  | β-actin | Mouse mAb | Santa Cruz | sc-81178 |
| **YAP** | YAP | Rabbit mAb | CST | #14075 |
|  | pYAP(Ser127) | Rabbit mAb | CST | #13008 |
| **EMT related proteins** | E-cadherin | Rabbit mAb | CST | #3195 |
|  | N-cadherin | Mouse mAb | CST | #14215S |
|  | Vimentin | Mouse mAb | Santa Cruz | sc-6260 |
|  | Twist | Mouse mAb | Santa Cruz | sc-81417 |
|  | Slug | Rabbit mAb | CST | #9585 |
|  | Snail | Rabbit mAb | CST | #3879 |
| **Rho GTPases** | Rac1 | Rabbit Pab | Santa Cruz | sc-95 |
|  | RhoA | Mouse mAb | Santa Cruz | sc-418 |
|  | Cdc42 | Rabbit mAb | Abcam | ab187643 |
|  | GEF-H1 | Rabbit mAb | Abcam | ab155785 |
| **Tight junction proteins** | Occludin | Mouse | Santa Cruz | sc-271842 |
|  | Claudin-5 | Rabbit | Santa Cruz | sc-28670 |
|  | ZO-1 | Rabbit | Santa Cruz | sc-10804 |
| **integrins** | integrin β1 | Mouse mAb | Santa Cruz | sc-374429 |
|  | integrin β3 | Mouse mAb | Santa Cruz | sc-46655 |
| **MMPs** | MMP-3 | Mouse mAb | Santa Cruz | sc-21732 |

**Table S2** PCR primers used for quantitative ChIP-PCR in this study.

| Gene Name | Sequence (5'-3') |
| --- | --- |
| CTGF | CTGF-F: 5'-GGTGCGAAGAGGATAGGGAAA -3'  CTGF-R: 5'-TGATCCTGACCCCTTGACACT-3' |
| CYR61 | CYR61-F: 5’-CTCACGACCCTCCAACTACCA-3’  CYR61-R: 5’-CTGGCTCCATTGCACCTTT-3’ |
| SNAI1 | SNAI1-F: 5'-CGCTCCGTAAACACTGGATAAG-3'  SNAI1-R: 5'-TTGACGAGGGAAACGCACA-3' |
| SNAI2 | SNAI2-F: 5'-GTGTTTTGTGGGAAATGGAGTG-3'  SNAI2-R: 5'-GTGAGGGTTTGTTCGGATGTAG-3' |
| AMOTL2 | AMOTL2-F: 5'-GGTTATTTGTGGTTCGCTTTGAT-3'  AMOTL2-R: 5'- CTGCTATGCCAGGAATGTGAGA-3' |
| ANKRD1 | ANKRD1-F: 5'-TGATTGAAACAGTAAGGAGCCA-3'  ANKRD1-R: 5'- CTCACCTAGACCCACTCAGGAT-3' |
| CDC42 | CDC42- F:5’-ATGTGGCTACAGCGAGGGAC-3’  CDC42-R:5’-CTGTATTCAGTCCGCCGTG-3’ |
| RHOA | RHOA-F:5’-AGATTTACTCACCACGAAGGG-3’  RHOA-R:5’-CTCTGGATGTGTTTCTGCCTC-3’ |
| RAC1 | RAC1-F:5’-CAAGCAGGAGTTAGTGACAAGG-3’  RAC1-R:5’-ACCGAGGACACGGAGGTT-3’ |
| METTL3 | METTL3-F:5’-CGTATTTGAAGACCTCTCGGAT-3’  METTL3-R:5’-CAACGGTGGTCCCCAATCT-3’ |
| METTL14 | METTL14-F:5’-GGGAAGAAGCCAAAAAATGC-3’  METTL14-R:5’-ACGCAGGTAACAGCCACAAC-3’ |
| FTO | FTO-F:5’-CATATCCTTGTGAGGCTGTTACTC-3’  FTO-R:5’-CATCCCGCTTCCTTGTTGT-3’ |
| WTAP | WTAP-F:5’-CAGAGATGTCAAGGAGGAACGA-3’  WTAP-R:5’-TGAGAACTTAGAAACCGATGCC-3’ |

**Table S3** PCR primers used for quantitative RT-PCR in this study.

| Gene Name | Sequence (5'-3') |
| --- | --- |
| GAPDH | Hgapdh-F: 5'-CTTTGGTATCGTGGAAGGACTC-3'  Hgapdh-R: 5'-GTAGAGGCAGGGATGATGTTCT-3' |
| β-actin | Hβ-actin-F: 5’-GTGGCCGAGGACTTTGATTG3’  Hβ-actin-R: 5’-CCTGTAACAACGCATCTCATATT3’ |
| YAP1 | YAP1-F: 5'-CAGAACCGTTTCCCAGACTACC-3'  YAP1-R: 5'-GCAGACTTGGCATCAGCTCCT-3’ |
| RAC1 | RAC1-F: 5'-GTCCCAACACTCCCATCATCC-3'  RAC1-R: 5'-TTACAGCACCAATCTCCTTAGCC-3' |
| RHOA | RHOA-F: 5'-GGAAGAAACTGGTGATTGTTGG-3'  RHOA-R: 5'-TTCAAAAACCTCTCTCACTCCA-3' |
| CDC42 | CDC42-F: 5'-TGACAGATTACGACCGCTGAGTT-3'  CDC42-R: 5'-AGGAGTCTTTGGACAGTGGTGAGT-3' |
| SNAI1 | SNAI1-F: 5'-CCCCAATCGGAAGCCTAA 3'  SNAI1-R: 5’-GCTGCTGGAAGGTAAACTCTG3’ |
| SNAI2 | SNAI2-F: 5'-AGCCAAATGACAAATAAAGTCC 3'  SNAI2-R: 5’-TCTCAATCTAGCCATCAGCAA 3’ |
| CTGF | CTGF-F: 5'-CTTCGGTGGTACGGTGTA3'  CTGF-R: 5’-GCTCTAATCATAGTTGGGTCT3’ |
| AREG | AREG-F: 5'-CGCTGCGAAGGACCAAT 3'  AREG-R:5’-CCCGAGGACGGTTCACTA 3’ |
| E-cadherin | E-cadherin-F: 5'-CCCCATACCAGAACCTCG 3'  E-cadherin-R:5’-TGGGTCGTTGTACTGAATGGT3’ |
| N-cadherin | N-cadherin-F: 5'-ATCCTACTGGACGGTTCGC3’  N-cadherin-R: 5’-CCTTGGCTAATGGCACTTG 3’ |
| ITGB1 | integrinβ1-F: 5'-CCTACTTCTGCACGATGTGATG  integrinβ1-R: 5'-CCTTTGCTACGGTTGGTTACATT |
| ITGB3 | Integrinβ3-F: 5'-ACGAAAATACCTGCAACC  Integrinβ3-R: 5'-CTGGCTCTTCTACCACATAC |
| EDN1 | EDN1-F: 5'-CACCTGGACATCATTTGGGTC 3’  EDN1-R: 5’-TTTTCACGGTCTGTTGCCTTT 3’ |
| ETS1 | ETS1-F: 5'-GTCAACCCAGCCTATCCA 3'  ETS1-R: 5’-GGCTCTGAGAACTCCGATG 3’ |
| F3 | F3-F: 5'-TGAAGGATGTGAAGCAGACG 3'  F3-R: 5’-CGAGGTTTGTCTCCAGGTAAG 3’ |
| HBEGF | HBEGF-F: 5'-CGTCGGTGGTGCTGAA 3'  HBEGF-R: 5’-CTTGTGGCTTGGAGGATAAA 3’ |
| AJUBA | AJUBA -F: 5'-TGTCACCGACTACCACAAA 3'  AJUBA -R: 5’-ATCACCCTCACGATGTCC 3’ |
| AMOTL2 | AMOTL2-F: 5'-GCCTATGTGGAGAAAGTGGAG 3'  AMOTL2-R: 5’-CTGCCTGTCTCTGCTGTGC 3’ |
| GTPBP4 | GTPBP4-F: 5'-AAGACTCAACGAAAGACTCCAAC3'  GTPBP4-R: 5’-CATCAAATCAGCATAGAACGG 3’ |
| METTL3 | METTL3-F:5'-ACAGAGTGTCGGAGGTGATT-3’  METTL3-R:5’-TGTAGTACGGGTATGTTGAGC-3’ |
| METTL14 | METTL14-F:5'-CCCATGTACTTACAAGCCGATAT-3’  METTL14-R:5’-CCCCATTTTCGTAAACACACTC-3’ |
| WTAP | WTAP-F:5'-CCTCTTCCCAAGAAGGTTCGAT-3’  WTAP-R:5’-GTTCCTTGGTTGCTAGTCGC-3’ |
| FTO | FTO-F:5'-AATAGCCGCTGCTTGTGAG-3’  FTO-R:5’-CCACTTCATCTTGTCCGTTG-3’ |

**Table S4** Silencing YAP target sequence.

| Clone Name | Location | Length | Target Sequence |
| --- | --- | --- | --- |
| shYAP21 | 857 | 21 | GCTCAGCATCTTCGACAGTCT |
| shYAP22 | 920 | 21 | GAGATGGCAAAGACATCTTCT |
| shYAP23 | 1051 | 21 | GCAGAATATGATGAACTCGGC |
| shYAP24 | 1103 | 21 | GCCATGACTCAGGATGGAGAA |
| TR001 |  | 19 | GTTCGCGCCGTAGTCTTA |

**Table S5** Gene lists involved in migration.

| **Gene Name** | **Gene Name** | **Gene Name** | **Gene Name** | **Gene Name** | **Gene Name** |
| --- | --- | --- | --- | --- | --- |
| ADGRA2 | PKN2 | CEP120 | CXCL8 | LYN | SYDE1 |
| ALX1 | PLXND1 | FHOD1 | DDR2 | MAPK14 | THBS1 |
| AMOTL1 | PRKX | HHEX | EDN1 | MDM2 | VEGFA |
| ARID5B | PTEN | MYH10 | ETS1 | MYADM | ZC3H12A |
| ARSB | PTK2B | SLC9A3R1 | F3 | NFE2L2 | ECM1 |
| CORO1B | ROBO1 | CDK5 | FER | NOTCH1 | MSN |
| EMP2 | SCARB1 | GLI3 | FERMT3 | PLAA | AJUBA |
| ID1 | SEMA3B | LAMB1 | FGF2 | PLK2 | AMOTL2 |
| LAMA5 | SEMA3F | DIXDC1 | FOXC2 | PTGS2 | EPHA2 |
| LRP5 | SEMA4C | ENG | GCNT2 | RPS6KB1 | FLCN |
| LTB4R2 | SRGAP2 | SNAI2 | HBEGF | RTN4 | GTPBP4 |
| MEGF8 | SYNE2 | ADAM17 | HMOX1 | SELENOK | JAG1 |
| NOV | TBX1 | C5AR1 | HSPA5 | SERPINE1 | KRT16 |
| PAXIP1 | TGFBR1 | CCL20 | HSPB1 | SNAI1 | MCTP1 |
| PDXDC1 | TNFSF12 | CRK | IL1A | SOD2 | NEXN |
| PHACTR4 | TWIST1 | CXCL2 | IL6 | SPAG9 | PHLDA2 |
| PKN1 | DAB2IP | CXCL3 | JUN | STX4 | RND3 |
| SDC4 | SERPINE2 | TIMP1 | TRIB1 | EFNB2 |  |

**Table S6** YAP positive target genes from HepG2 VS FSS-HepG2.

| **Gene Name** | **Gene Name** | **Gene Name** | **Gene Name** | **Gene Name** | **Gene Name** |
| --- | --- | --- | --- | --- | --- |
| AJUBA | FST | ABHD10 | DIS3L | METTL13 | TBC1D4 |
| AKAP12 | GADD45A | ADAMTS6 | DKK1 | MRPL24 | TDP1 |
| AMOTL2 | GADD45B | ARSJ | DPH5 | MTFP1 | TIMELESS |
| ANKRD1 | GNL2 | BASP1 | EID2 | MYC | TK1 |
| ANKRD33B | GNL3 | BCAT1 | ERLIN1 | NEURL1B | TMEM106C |
| ARNTL2 | GPATCH4 | C1QBP | FJX1 | NUF2 | TMEM200B |
| ASAP1 | GRPEL1 | C4BPB | GEMIN4 | POC1A | TOP2A |
| CRY1 | GTPBP4 | CCDC137 | GINS1 | POLA2 | TRIM14 |
| CYP20A1 | HBEGF | CCNA2 | GPN3 | POLH | TRMT5 |
| DDX21 | HSPA9 | CDCA4 | HAUS4 | PSRC1 | TROAP |
| DNTTIP2 | LARP4 | CDKN2AIPNL | HIST1H3B | RALGPS2 | TSEN2 |
| EDN1 | LSG1 | CENPF | KATNB1 | RPUSD2 | TUBB6 |
| EIF5A2 | MRPL18 | CEP57 | KIF18B | RTN4IP1 | TUBG1 |
| ETS1 | NAA25 | CKAP2L | KNTC1 | SEPT11 | UBE2E2 |
| F3 | NEDD4L | COL12A1 | LIMA1 | SERTAD4 | ZWILCH |
| FAM57A | NUP50 | CYR61 | MCM3 | SF3B3 |  |
| FOSL1 | ODC1 | DARS2 | MEST | SNAI2 |  |

**Table S7** YAP-targeted genes involved in migration.

| **Gene Name** |
| --- |
| EDN1 |
| ETS1 |
| F3 |
| HBEGF |
| AJUBA |
| AMOTL2 |
| GTPBP4 |
| SNAI2 |
